# Genetic manipulation of *cys*H/*cys*J in *Citrobacter* sp. XT1-2-2 enhanced cadmium immobilization by regulating metabolic pathways

**DOI:** 10.1128/aem.00856-25

**Published:** 2025-08-21

**Authors:** Zhudong Liu, Wei Cheng, Yilu Li, Shiping Shan, Shandong Wu, Xiaowu Wei, Hua Yang, Min Zhang, Dongxia Du

**Affiliations:** 1 Hunan Institute of Microbiology, Hunan Academy of Agricultural Sciences 441102, Changsha, Hunan, China; 2 Yuelushan Laboratory, Changsha, Hunan, China; 3 Hunan Engineering and Technology Research Center of Agricultural Microbiology Application, Changsha, Hunan, China; Colorado School of Mines, Golden, Colorado, USA

**Keywords:** *Citrobacter *sp. XT1-2-2, cadmium immobilization, CdS, sulfur metabolism, hydrogen sulfide

## Abstract

The *Citrobacter* sp. XT1-2-2 strain has emerged as a promising candidate for cadmium immobilization; however, the genetic basis underlying its sulfur-mediated bioremediation mechanisms remains inadequately understood. To address this gap, we concentrated on two pivotal genes, *cys*H and *cys*J, within the sulfate assimilation pathway. We constructed *Citrobacter* sp. XT1-2-2-::APS and *Citrobacter* sp. XT1-2-2-::SiR strains with overexpression of *cys*H or *cys*J for functional characterization. Transmission electron microscopy demonstrated a significant enhancement in the biosynthesis of CdS nanoparticles in both overexpression strains. This increase was attributed to the elevated production of hydrogen sulfide. Complementary physicochemical analyses, including Fourier transform infrared spectroscopy, X-ray photoelectron spectroscopy, and powder X-ray diffraction, further indicated that overexpression of *cys*H or *cys*J modified functional groups on the surface and enhanced the efficiency of sulfur metabolism. Microcosm experiments demonstrated that the contents of Cd^2+^ in the roots, culms, leaves, and grains inoculated with overexpression strains were significantly lower than those observed in the wild-type strain. These findings establish a critical role for *cys*H/*cys*J-mediated metabolic pathway regulation in cadmium immobilization. They provide a theoretical foundation for the exploration of novel bacterial-assisted techniques, marking a breakthrough in environmental biotechnology from fundamental research to the engineering application of genetically engineered bacteria.

**IMPORTANCE:**

The presence of cadmium in paddy soil poses a significant concern, primarily due to its potential threat to food safety and public health within the soil-plant system and the broader food chain. The genes *cys*H and *cys*J were overexpressed under the regulation of the erythromycin promoter within the sulfate assimilation pathway in the *Citrobacter* sp. XT1-2-2 strain. The resulting overexpression strains (XT1-2-2-::APS and XT1-2-2-::SiR) exhibited enhanced biosynthesis of CdS nanoparticles, attributed to increased hydrogen sulfide production. Compared to the wild-type strain, cadmium concentrations in the grains of XT1-2-2-::APS and XT1-2-2-::SiR were reduced by 36.49% and 62.56%, respectively. Furthermore, the residual cadmium content in the soil was elevated by 36.36% (*P* < 0.01) and 27.27% (*P* < 0.01), respectively. These results provided a theoretical foundation for the exploration of novel bacterial-assisted techniques aimed at cadmium remediation, marking a breakthrough in environmental biotechnology from fundamental research to the engineering application of genetically engineered bacteria.

## INTRODUCTION

As is widely recognized, cadmium pollution has emerged as a significant environmental concern. This issue primarily stems from mining activities and various industrial processes, the excessive application of chemical fertilizers in agricultural soils, and water contamination (1). A soil survey revealed that 19.4% of China’s total arable land surpasses the regulatory thresholds for soil pollution (2). The primary inorganic contaminants identified in the soil consist of cadmium (Cd), lead (Pb), arsenic (As), and mercury (Hg). Among these contaminants, cadmium represents the largest proportion of inorganic pollutants (3). The presence of cadmium in paddy soil poses a significant concern, primarily due to its potential threat to food safety and public health within the soil-plant system and the broader food chain (4, 5). Numerous traditional techniques are employed to mitigate cadmium pollution in the soil. These methods encompass landfilling, electrodeposition, chemical leaching, and a range of physical and chemical techniques. However, these interventions have resulted in irreversible changes to soil quality and have disrupted the ecological balance as well as bacterial diversity within the soil (6). Addressing cadmium contamination in agricultural soils presents a significant challenge; therefore, it is crucial to identify a more effective, cost-efficient, and convenient strategy for remediating cadmium pollution from both economic and practical perspectives (7).

In recent years, the utilization of microbial technology for the remediation of cadmium pollution has emerged as a prominent area of research and attracted widespread attention from scientists worldwide (8). Microorganisms present in the soil have the capacity to effectively mitigate cadmium pollution and can thrive in agricultural soil characterized by relatively high concentrations of heavy metals (9). The methods of microbial remediation primarily encompass biological adsorption and biological precipitation. Biological adsorption entails the utilization of amino acids, along with -COOH, -OH, and -SH groups that are present on the surfaces of microbial strains (10). In contrast, biological precipitation occurs as a result of interactions between heavy metal ions and polymers, phosphates, or secondary metabolites produced via these strains (11).

So far, a considerable number of scientists have concentrated on the immobilization of cadmium in soils contaminated with cadmium by microbial remediation techniques (12). These microorganisms can effectively reduce cadmium bioavailability through various microbial metabolic processes (13, 14). Screening and isolating strains with beneficial functions for cadmium immobilization and enhancing plant growth has emerged as a prominent research focus in recent years. A report highlighted the successful screening and identification of a novel bacteria with high cadmium-tolerant ability in the cadmium-contaminated soil, and these bacteria exhibited a remarkable capacity to mitigate the biotoxicity associated with cadmium (15). Mathivanan et al. (16) elucidated that the synthesis and compositional analysis of exopolysaccharides produced through *Bacillus cereus* KMS3-1 demonstrated a remarkable capacity for metal adsorption. Suksabye et al. (17) demonstrated that biochars and microorganisms can be effectively utilized to address the issue of cadmium pollution in paddy soils. Previous research has shown that a variety of functional microorganisms found in the soil possess the ability to remediate cadmium pollution in paddy soils (18). China is recognized as one of the world’s leading rice-producing nations. Consequently, it has become increasingly imperative to investigate and develop microbial remediation strategies for cadmium-contaminated rice to mitigate the daily intake of heavy metals by humans (19).

The *Citrobacter* sp. strain has been documented to possess the capability of reducing heavy metals in both soil and industrial wastewater (20). Liao et al. (21) elucidated the mechanisms employed by *Citrobacter* sp. for the removal of 2,4,6-trinitrotoluene (TNT). Singh et al. (22) demonstrated that *Citrobacter* sp. IITISM25 could be effectively utilized in the remediation of mercury(II) stress conditions. Shan et al. (23) isolated a *Citrobacter* sp. XT1-2-2 from soils heavily contaminated with cadmium, and the strain demonstrates a remarkable capacity to diminish the bioavailability of cadmium within the soil and to impede its absorption by rice plants. The *Citrobacter* sp. strain, a type of Gram-negative bacterium with cadmium tolerance and metabolic activity, has been demonstrated to reduce cadmium uptake in rice through multiple mechanisms, thereby exhibiting significant application potential (24). However, the mechanisms underlying cadmium immobilization by *Citrobacter* sp. remain inadequately understood. It is imperative to elucidate the genetic basis underlying its sulfur-mediated bioremediation mechanisms in cadmium-contaminated paddy soil.

The objective of this investigation was to augment the expression levels of critical enzyme genes within the sulfate metabolic pathway of the *Citrobacter* sp. XT1-2-2 strain through gene editing techniques, thereby enhancing the strain’s capacity to promote plant growth while simultaneously mitigating cadmium absorption. The *cys*H and *cys*J in *Citrobacter* sp. XT1-2-2 is a key component of the bacterial sulfate assimilation and reduction pathway (Assimilatory Sulfate Reduction, ASR), responsible for converting inorganic sulfate into hydrogen sulfide (H_2_S) required for cysteine synthesis. They interact closely with components of other sulfur assimilation pathways, collectively forming a precisely regulated metabolic network. Our focus has been directed towards two essential enzymes that play a pivotal role in the sulfate reduction pathway: adenosine 5'-phosphosulfate reductase (CysH) and sulfite reductase (CysJ). In *Citrobacter* sp. XT1-2-2, these genes not only operated in isolation but also deeply embedded in the network of the sulfur assimilation and reduction pathway, closely collaborating with upstream activating enzymes, downstream reducing partners, reducing power supply systems, and global/pathway-specific regulatory factors to jointly complete the core task of converting inorganic sulfate into bioavailable organic sulfur (cysteine). By employing strategic genetic engineering methodologies utilizing the erythromycin resistance promoter (PermE) (25), we sought to elevate the expression of these significant enzyme genes, *cys*H and *cys*J. This approach enabled us to systematically investigate their synergistic effects on three critical biological processes: cadmium biosorption dynamics, the synthesis of CdS nanoparticles, and extracellular polymeric substance-mediated biofilm formation. The phenotypic consequences of enzymatic overexpression were thoroughly characterized through ultrastructural analysis utilizing transmission electron microscopy (TEM). This was further complemented by multi-technique validation, which included Fourier transform infrared spectroscopy (FT-IR) for the purpose of identifying functional groups, X-ray photoelectron spectroscopy (XPS) for the insightful profiling of surface chemistry, and powder X-ray diffraction (p-XRD) for crystallographic characterization. To elucidate the physiological mechanisms underlying cadmium mitigation, we conducted microcosm experiments quantifying cadmium distribution patterns across rice tissues (roots, culms, leaves, and grains). This multidimensional approach not only elucidates the molecular mechanisms governing sulfate metabolism in the context of cadmium detoxification but also establishes a proof-of-concept framework for engineering microbial systems with enhanced bioremediation capabilities through targeted optimization of metabolic pathways.

## MATERIALS AND METHODS

### Experimental materials

The *Citrobacter* sp. XT1-2-2 was isolated from paddy soils contaminated with cadmium in Hunan, China (28°01′ N, 113°34′ E). The XT1-2-2 strain was cultivated at a temperature of 30°C in SRB medium, under conditions of static culture. The growth curve of strain XT1-2-2 indicated that the growth rate was relatively slow during the initial 48 hours, which corresponded to a period of growth delay. From 48 to 72 hours, the strain entered the logarithmic growth phase, followed by a stability period from 72 to 96 hours. Subsequently, it began to transition into a decline phase. The strains, plasmids, and primers utilized in this study are kindly detailed in Table S1. The formulation of the SRB liquid medium is detailed as follows: 0.5 g/L Na_2_SO_4_, 0.2 g/L CaCl_2_·2H_2_O, 2.7 g/L MgSO_4_·7H_2_O, 0.5 g/L K_2_HPO_4_, 1.3 g/L NH_4_Cl, 6 g/L sodium lactate, 1.0 g/L ascorbic acid, 1.4 g/L yeast extract, and 0.2 g/L FeSO_4_·(NH_4_)_2_SO_4_·6H_2_O (as an indicator). Agar, at a concentration of 20.0 g/L, was incorporated into the formulation for the SRB agar plates.

### Refinement of the overexpression strains

The pBBR1MCS-2 plasmid was utilized in the construction of overexpression strains (26). The primers utilized in this study are presented in Table S1. The pBBR1MCS-2 and PermE-*cys*H recombinant fragments were subjected to double digestion using the restriction enzymes *Xba*I and *EcoR*I (Fig. S1). The linearized pBBR1MCS-2 and the PermE-*cys*H recombinant fragment were ligated using T4 ligase to generate the recombinant vector pBBR1MCS-PermE-*cys*H. The successful construction of the recombinant vector pBBR1MCS-PermE-*cys*H was validated through double enzyme digestion using *Xba*I and *EcoR*I on the recombinant vector (Fig. S2). The recombinant vector pBBR1MCS-PermE-*cys*H was successfully transformed into the competent cells of the XT1-2-2 strain through electroporation, and positive colonies were successfully obtained through screening with kanamycin at a concentration of 50 µg/mL (Fig. S3) (27, 28). The construction of the XT1-2-2-::APS strain has been successfully validated through PCR and sequencing techniques. The construction of the XT1-2-2-::SiR strain was aligned with the process utilized for the XT1-2-2-::APS strain (Fig. S4 to S6).

### Characterizations of overexpression strains

The wild-type strain, along with XT1-2-2-::APS and XT1-2-2-::SiR variants, was kindly cultivated in liquid SRB medium under both conditions: the absence and presence of 100 mg/L Cd²^+^. The bacterial cells were washed five times with a phosphate-buffered saline (PBS) solution. Subsequently, the culture solutions underwent centrifugation at 12,000 r/min for a duration of 15 minutes at a temperature of 4°C, facilitating the collection of the resultant bacterial precipitates. Then, remove the supernatant carefully and gently introduce 1.5 mL of isoglutaraldehyde electron microscopy fixative buffer along the inside wall of the tube, ensuring that the bacterial precipitate remains undisturbed. The strains were subsequently stored at 4°C for further analysis. The morphological attributes and intracellular sequestration of Cd^2+^ within the strains were investigated utilizing transmission electron microscopy (TEM) (H-7650, Hitachi, Japan) (29). The X-ray photoelectron spectroscopy (XPS) of the strains was conducted with an ESCALAB 250Xi instrument (Thermo Fisher Corporation, USA) (30). The powder X-ray diffraction (p-XRD) of the strains was performed (31). Fourier transform infrared spectroscopy (FT-IR) spectra of the strains were recorded utilizing a Bruker Tensor 27 Fourier transform infrared spectrometer (Bruker Optics, Germany) (32, 33).

### Analysis of cadmium adsorption

The strains were grown in SRB liquid medium supplemented with 100 mg/L of Cd^2+^. Supernatant samples were collected at various time points, specifically at 0, 20, 40, 60, 80, 100, and 120 hours during the cultivation period. The concentration of Cd^2+^ in the collected supernatants was analyzed using an inductively coupled plasma mass spectrometer (ICP-MS; Thermo Fisher, iCAP MSX ICP-MS) (34).

### Analysis of enzyme activity

The wild-type, XT1-2-2-::APS, and XT1-2-2-::SiR strains were harvested separately at 48 hours (12,000 r/min for 15 minutes at 4°C) and subsequently washed three times with PBS (10 mmol/L, pH 7.0, stored at 4°C). The strains were resuspended in 200 µL of lysozyme solution (0.2 g/mL), followed by the addition of 600 µL of lysis buffer to each individual tube. The whole-cell proteins were then extracted using ultrasonic fragmentation with a JY92 ultrasonic cell grinder. The composition of the protein extraction lysis buffer used for protein extraction includes 8 mol/L urea, 2 mol/L thiourea, 5% (w/v) CHAPS, 150 mmol/L NaCl, and 50 mmol/L Tris-HCl (pH = 7.4). Additionally, it contains 1% NP-40, 1% Triton X-100, and freshly added 1 mmol/L PMSF, and 6 µL of protease inhibitor cocktail (EDTA-Free, 100 × in DMSO). The results were recorded at an optical density of 450 nm using a microplate reader within 15 minutes. The APS reductase activity was assessed utilizing the Quickcheck SRBTM kit. The SiR reductase activity was determined using the SiR reductase kit (35).

### Analysis of biofilm formation

The suspensions of the wild-type, XT1-2-2-::APS, and XT1-2-2-::SiR strains were inoculated into 80 mL of SRB medium at a dilution ratio of 1:50. The cultures were then incubated anaerobically at a temperature of 30°C. Subsequently, the cultures were transferred into 96-well plates, each containing a refined SRB liquid medium at a precise dilution of 1:100 during the logarithmic growth phase, with each well receiving 100 µL. The plates were then subjected to anaerobic static culture at 30°C. Afterwards, the contents of each well were aspirated and washed with ddH_2_O, repeating this process four times for a total of five washes. Following the washing steps, a total of 120 µL of a 0.2% crystal violet solution was added to each well for staining, and this process was allowed to proceed for a duration of 15 minutes. Finally, the wells were aspirated again and cleaned twice to remove excess stain. The stained biofilm plates were allowed to air-dry, after which the biofilms were dissolved in 200 µL of 30% acetic acid. This solution was then permitted to stand undisturbed for a duration of 15 minutes. Following the process of absorption and mixing, 150 µL aliquots from each sample were carefully transferred to a pristine 96-well plate. Subsequently, all samples underwent measurement at a precise wavelength of 550 nm utilizing a microplate reader (36, 37).

### Microcosm experiments

The soil samples used in the microcosm experiments were collected from a cadmium-contaminated site located in Yiyang, Hunan Province, China, where the cadmium concentration was measured at 0.39 mg/kg. The primary physicochemical parameters of the obtained soil are shown in Table S2. The microcosm experiments consisted of three distinct treatments: soil amended with the wild-type XT1-2-2 (Soil+XT1-2-2), soil amended with the XT1-2-2-::APS strain (Soil+::APS), and soil amended with the XT1-2-2-::SiR strain (Soil+::SiR). Each treatment was replicated three times and arranged randomly. For each experimental treatment, a total of 5.0 kg of soil was placed into a pot characterized by a diameter of 50 cm and a depth of 30 cm. The bacterial culture solution was subjected to centrifugation at 11,000 r/min for a duration of 15 minutes. Following this, the pellet was washed five times with sterilized double-distilled water (ddH_2_O). Each strain inoculum, prepared to a density of 1.0 × 10^8^ CFU/mL, was achieved by resuspending the strain pellets in ddH_2_O. The resultant inoculum was then gradually and evenly distributed across the surface of the soil. Subsequently, the bacteria were thoroughly mixed with 5.0 kg of soil and maintained at 70% water-holding capacity for a duration of five days (38–40). The rice plants were transplanted into pots, with four seedlings allocated per pot. These planted pots were placed in a greenhouse, where the average temperature ranged from 25 ± 3.5°C during the day to 21 ± 3.5°C at night, accompanied by an average illumination of 12 hours per day. A water-holding capacity of 75% was maintained throughout the growth period. The roots, culms, leaves, and polished grains were separated at the time of rice harvest. They were then oven-dried at 85°C until a constant weight was achieved and subsequently ground to a particle size of less than 0.25 mm. Soil samples from each treatment were collected post-harvest, air-dried, and ground to a particle size of 0.15 mm.

### Analysis of soil samples

The five geochemical forms of cadmium in soil are as follows: water-soluble and exchangeable state (SE), carbonate-bound state (WSA), iron/manganese oxide-bound state (OX), organic matter-bound state (OM), and residual component (RES). The sequential extraction method was employed to assess the changes in the speciation of cadmium in soil. The detailed extraction procedures for these five geochemical forms within the soil remain consistent with those outlined in previous studies. The ICP-MS was employed to assess the concentration of cadmium in the soil.

### Analysis of plant samples

After harvesting the rice plants at the mature stage in each treatment of the microcosm experiments, the roots, culms, leaves, and grains were carefully separated. Then, dry all samples in an oven at 75°C. The concentrations of cadmium were determined using ICP-MS. Additionally, the activities of six enzymes in mature leaves were measured. These enzymes included superoxide dismutase (SOD), glutathione enzyme (GSH), L-ascorbic acid (Vc), catalase (CAT), malate dehydrogenase (MDA), and peroxidase (POD). The leaf samples were ground into a fine powder and sent to Qingdao Kechuang Quality Testing Co., Ltd. for analysis.

### Statistical analysis

In order to guarantee the statistical validity, three replications of each experiment were carried out. Student’s *t*-tests were used to compare two groups. The mean ± standard deviation (SD) is used to display the experimental results. *P* < 0.05 was considered statistically significant for all tests. Tukey’s post hoc test was performed after one-way analysis of variance (ANOVA) for the comparisons among different groups. The IBM SPSS Statistics version 24.0 was used to perform all the statistical analyses.

## RESULTS

### Analysis of enhanced sulfide production

The overexpression strains were successfully generated through electroporation into the protoplasts of *Citrobacter* sp. XT1-2-2 (Fig. S3 and S6). The wild-type, XT1-2-2-::APS, and XT1-2-2-::SiR strains were cultured under identical conditions. After 48 hours, morphological changes in the bacterial colonies on the medium were observed. The colonies of all three strains exhibited a black coloration, indicating that these strains produced sulfide ions (S^2-^) which subsequently reacted with ferrous ions (Fe^2+^) present in the solid medium (composed of Fe^2+^ and ascorbic acid) to form black precipitates of iron sulfide (FeS). We observed that the strains with overexpression exhibited a darker coloration compared to the wild-type bacteria (Fig. S11). This phenomenon may be attributed to the overexpression of *cys*H and *cys*J.

### Analysis of intracellular CdS biosynthesis

**Fig 1:**
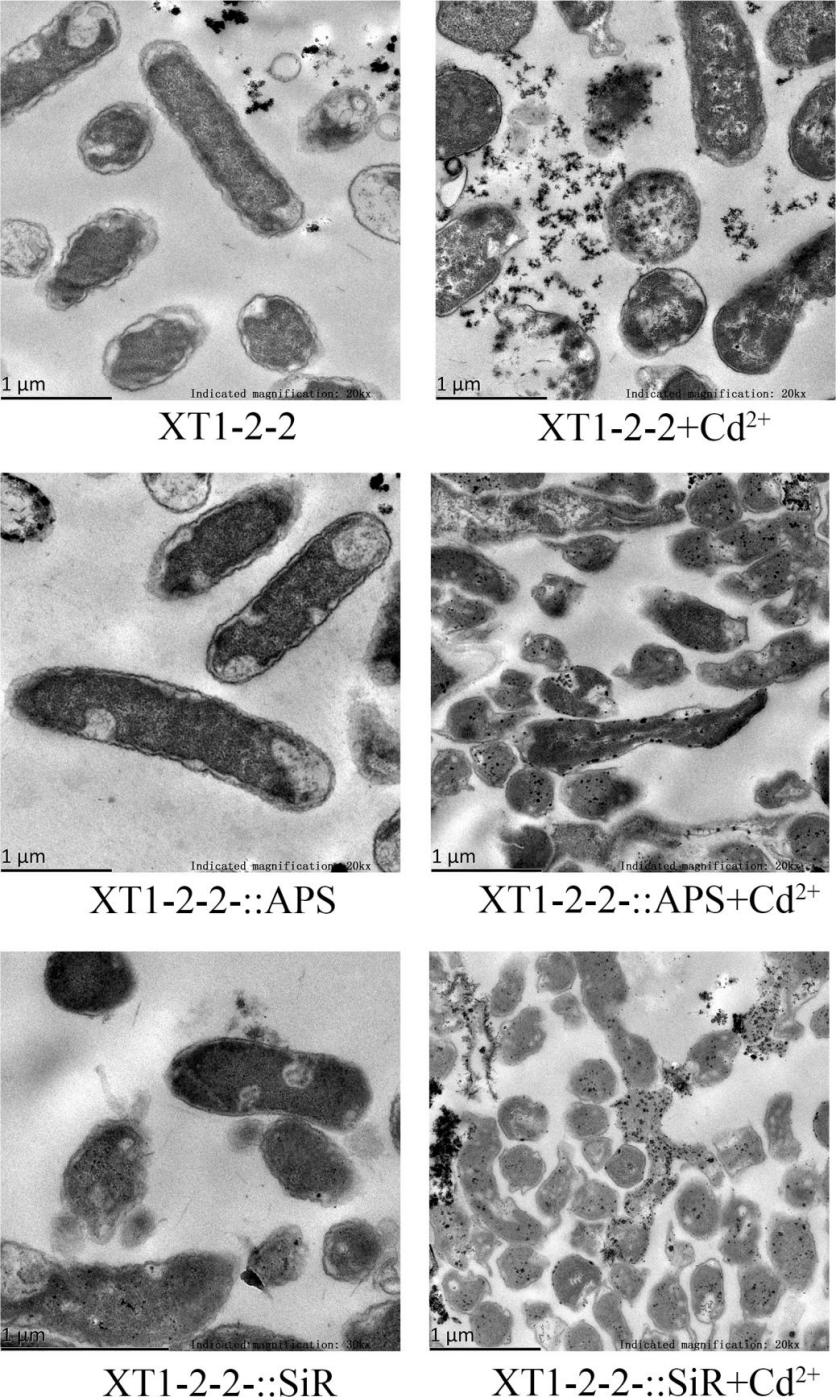
Transmission electron microscopy (TEM) analysis of the XT1-2-2, XT1-2-2-::APS, and XT1-2-2-::SiR strains.

To investigate the alterations in bacterial morphology resulting from gene overexpression, TEM images of the wild-type, XT1-2-2-::APS, and XT1-2-2-::SiR strains were examined (Fig. 1). The size of the wild-type strain exposed to 100 mg/L Cd^2+^ was observed to be smaller than that of the wild-type strain without Cd^2+^. Black particles formed in wild-type strains containing 100 mg/L Cd^2+^ were predominantly located outside the bacteria. In contrast, the black particles generated in XT1-2-2-::APS and XT1-2-2-::SiR strains exposed to 100 mg/L Cd^2+^ were primarily found within the bacterial cells, resulting in significant morphological changes to the bacteria. The presence of black particles indicates the biosynthesis of CdS in the strains.

### Analysis of enhanced cadmium removal efficiency

**Fig 2:**
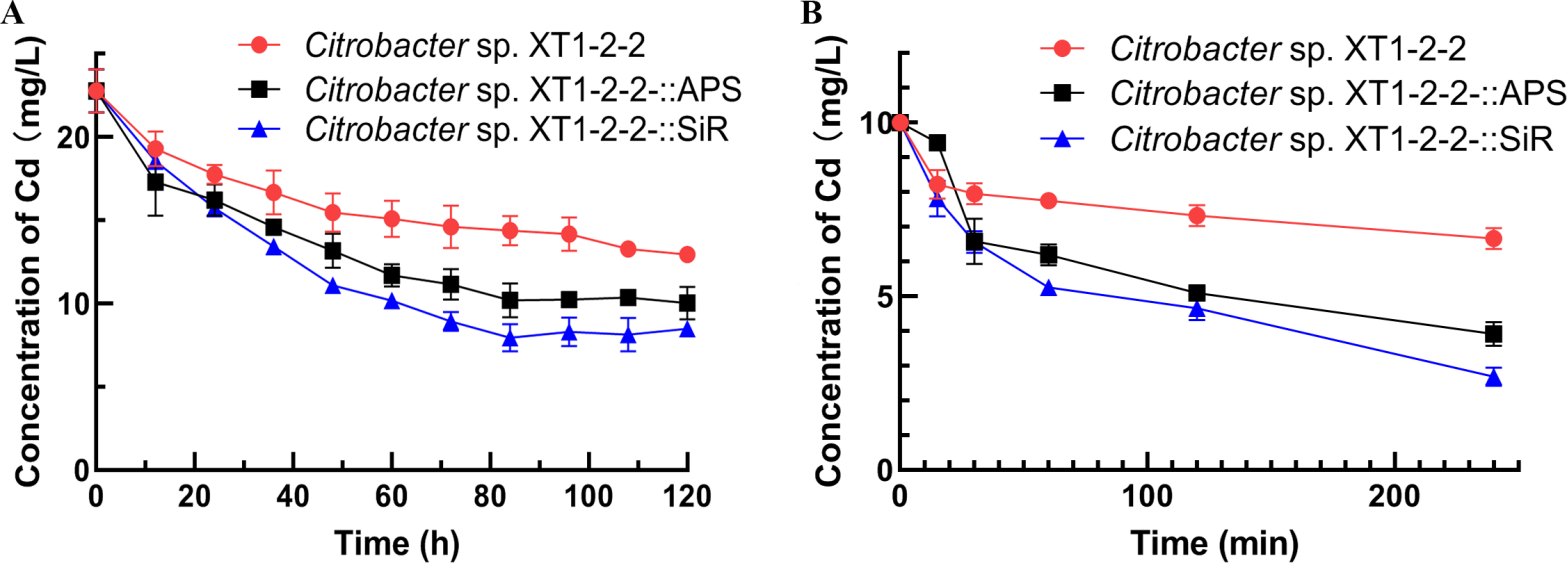
The differences in cadmium adsorption efficiency among the XT1-2-2, XT1-2-2-::APS, and XT1-2-2-::SiR strains in liquid SRB medium are presented, as indicated by the cadmium concentration in the supernatant of culture medium containing 100 mg/L Cd^2+^. Standard errors for results obtained from three replicates are indicated by error bars.

The cadmium concentration in the supernatant of the wild-type, XT1-2-2-::APS, and XT1-2-2-::SiR strains containing 100 mg/L Cd^2+^ was measured over a period of 120 hours (Fig. 2). The cadmium levels in these strains began to decline after 60 hours and continued to decrease gradually after reaching 72 hours. In comparison to the wild-type strain, the overexpression strains exhibited a significant reduction in cadmium content, achieving their lowest levels at 84 hours. Specifically, compared to the wild-type, the cadmium concentrations in XT1-2-2-::APS and XT1-2-2-::SiR were significantly reduced by 55.97% and 65.08%, respectively.

### Analysis of elevated intracellular CdS accumulation

**Fig 3:**
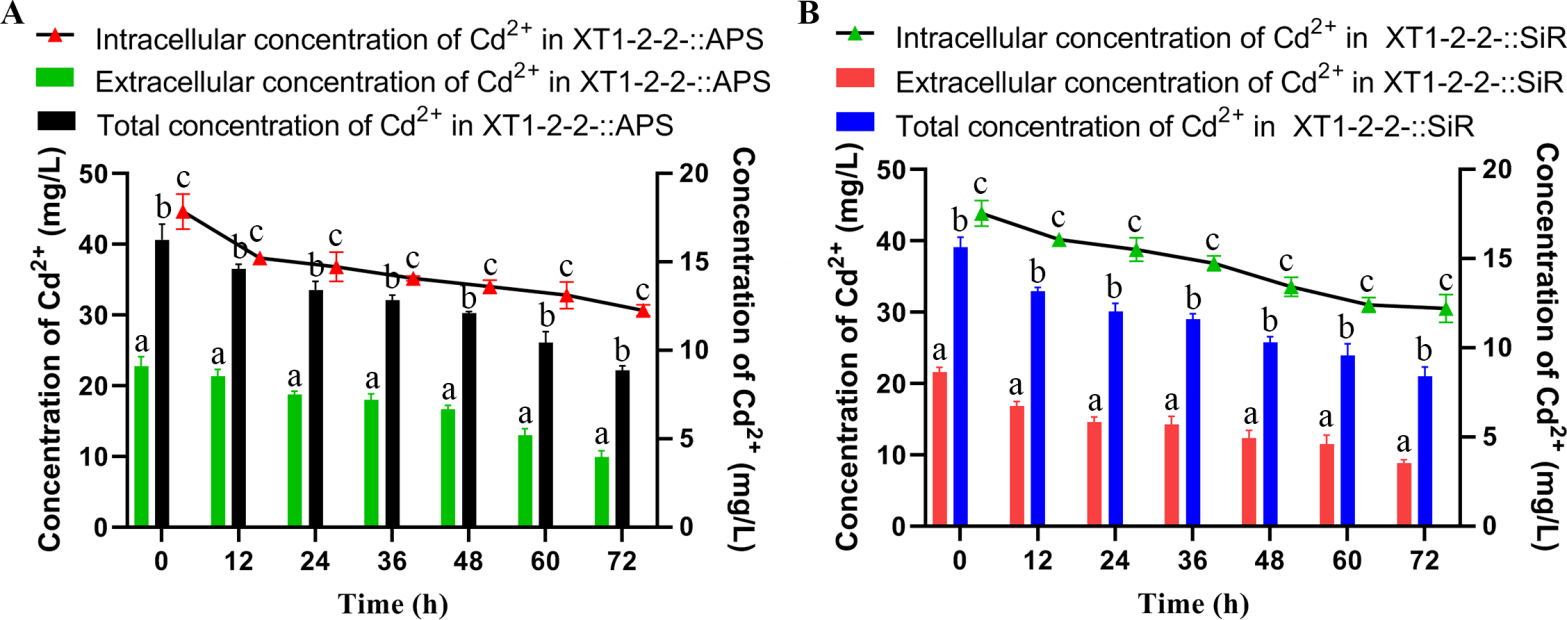
Distribution of intracellular and extracellular cadmium in the wild-type, XT1-2-2-::APS, and XT1-2-2-::SiR strains. (**A**) Changes in the concentrations of intracellular and extracellular cadmium accumulation in the XT1-2-2-::APS strain over a 72-hour period. (**B**) Changes in the concentrations of intracellular and extracellular cadmium accumulation in the XT1-2-2-::SiR strain over a 72-hour period.

To investigate the factors contributing to the alterations in the formation position of CdS resulting from the overexpression of *cys*H and *cys*J, we measured Cd^2+^ concentrations both intracellularly and on the surface of bacterial cells (Fig. 3). Our analysis revealed that after 60 hours, the intracellular concentration of Cd^2+^ in the XT1-2-2-::APS and XT1-2-2-::SiR strains decreased by 26.5% and 29.3%, respectively. In contrast, at the same time point, the extracellular concentration of Cd^2+^ in these strains diminished by 43.0% and 46.6%, respectively. Furthermore, our previous study indicated that within wild-type strains, Cd^2+^ accounted for approximately 10% of total cadmium levels inside cells (23). In comparison, for XT1-2-2-::APS and XT1-2-2-::SiR strains, intracellular percentages of Cd^2+^ relative to total cadmium were recorded at 50.2% and 51.9%, respectively. These results suggest that overexpression of *cys*H and *cys*J leads to an increased accumulation of CdS within bacterial cells.

### Analysis of biofilm formation differences

**Fig 4:**
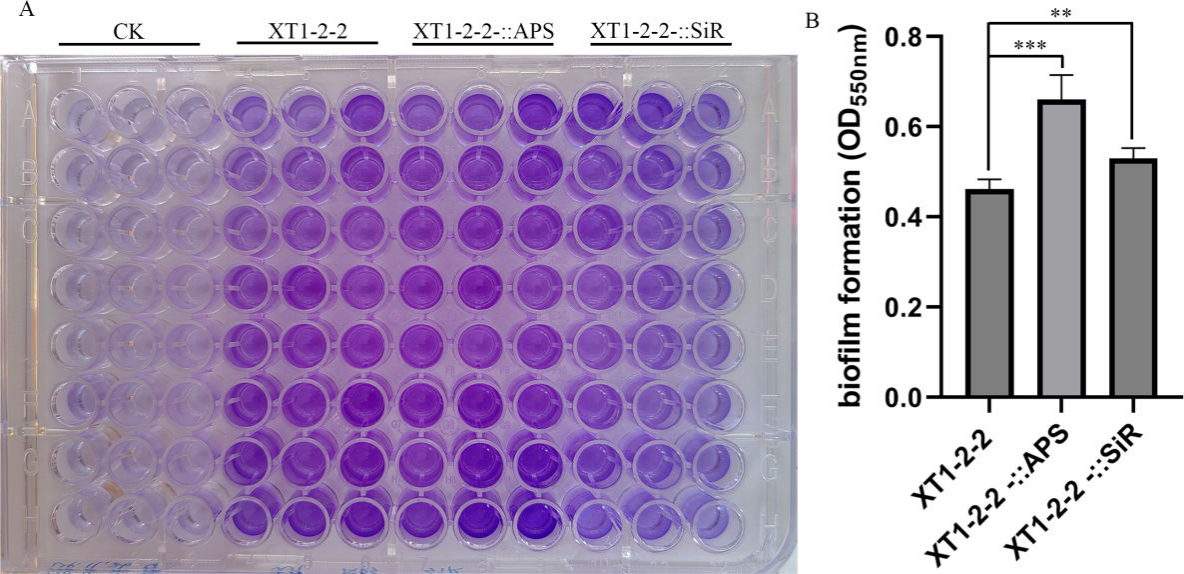
The differences in biofilm formation are presented. (**A**) Biofilm formation on 96-well plate-attached surfaces under static conditions for the wild-type, XT1-2-2-::APS, and XT1-2-2-::SiR strains. (**B**) Bacterial cell aggregation observed in the wild-type, XT1-2-2-::APS, and XT1-2-2-::SiR strains. Standard errors of the results from three replicates are indicated by error bars. Statistical significance is denoted by *, **, and *** for *P* < 0.05, *P* < 0.01, and *P* < 0.001, respectively.

Compared to the wild-type strain, the cell aggregation in XT1-2-2-::APS and XT1-2-2-::SiR increased by 43.05% and 14.6%, respectively, when cultivated in 96-well plates under static conditions. The XT1-2-2-::APS strains demonstrated the highest rate of cell aggregation, as illustrated in Fig. 4.

### Analysis of enzyme activity differences

**Fig 5:**
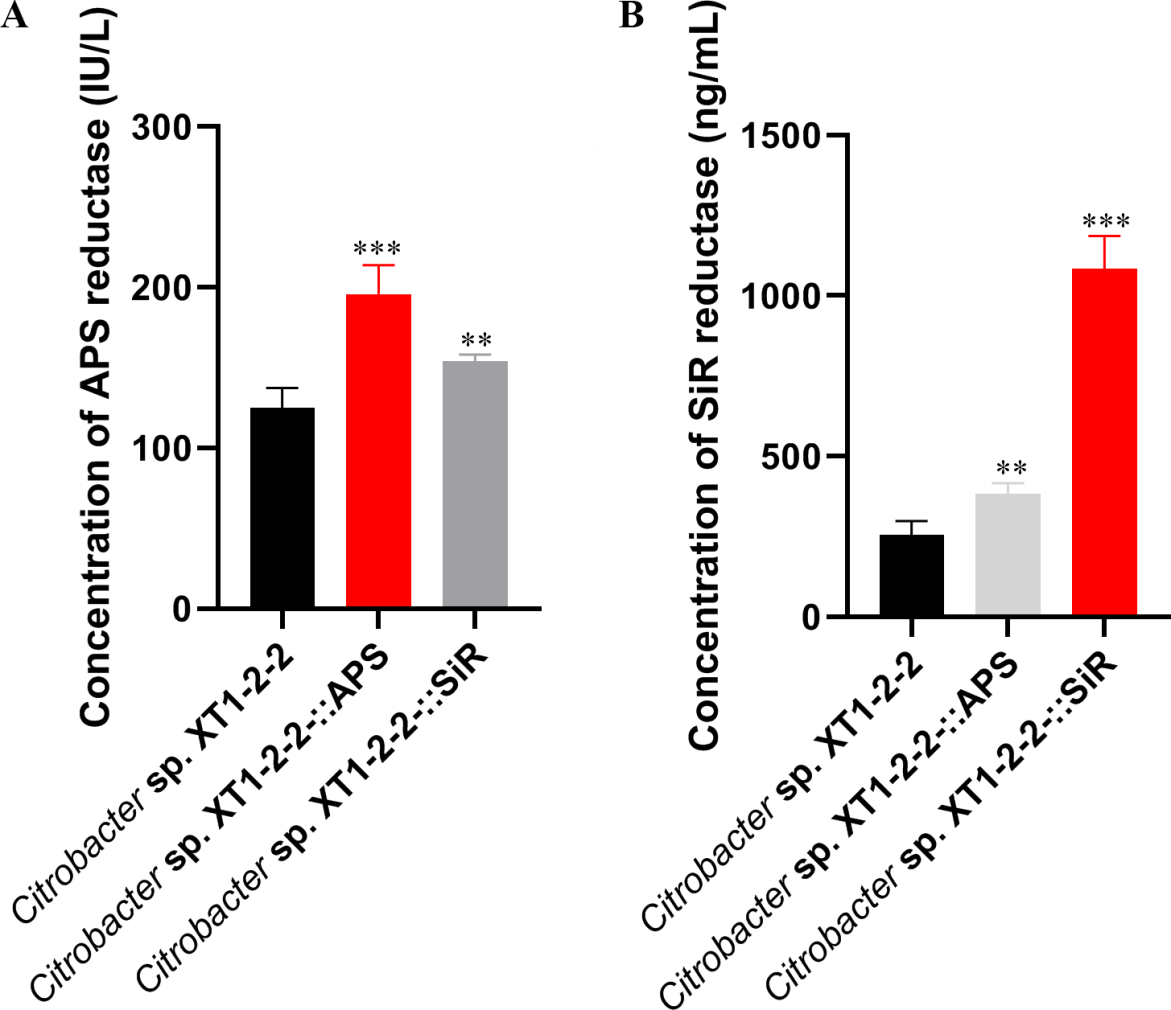
(**A**) Analysis of APS reductase activity in the wild-type, XT1-2-2-::APS, and XT1-2-2-::SiR strains. (**B**) Examination of SiR reductase activity in the wild-type, XT1-2-2-::APS, and XT1-2-2-::SiR strains. Data are presented as means ± standard deviations. The standard errors for the results obtained from three replicates are represented by error bars. Asterisks (*, **, ***) denote statistical significance levels of *P* < 0.05, *P* < 0.01, and *P* < 0.001, respectively.

To compare the changes in the activity of sulfate (APS) reductase and sulfite (SiR) reductase among these strains, we performed an enzyme activity assay (Fig. 5). The results revealed that the APS reductase activity increased by 56.65% and 23.25% for XT1-2-2-::APS and XT1-2-2-::SiR, respectively. Additionally, the SiR reductase activity exhibited increases of 41.09% for XT1-2-2-::APS and a remarkable 326.55% for XT1-2-2-::SiR.

### FT-IR difference analysis

**Fig 6:**
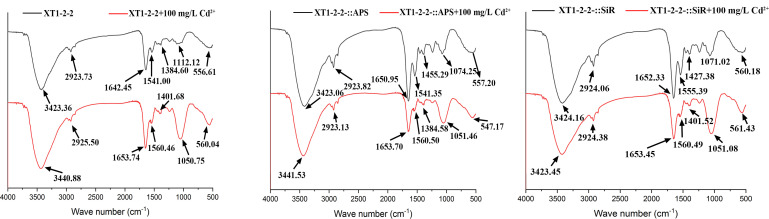
Analysis of Fourier transform infrared spectroscopy (FT-IR) spectra for the wild-type, XT1-2-2-::APS, and XT1-2-2-::SiR strains.

The interplay between the chemical functional groups present on the surface of the strains and Cd^2+^ may be influenced by the overexpression of *cys*H and *cys*J. The characteristic peaks observed for these strains are presented in Fig. 6. Through our previous studies, we identified the involvement of several functional groups, including the stretching vibrations of N-H, O-H, -NH, and carbonyl (23). Additionally, we observed stretching vibrations of C-O in carboxyl and S = O groups, -SO_3_ groups, and halogens or alkenes. The carbonyl stretching vibrations associated with amide groups and -NH distortion bands range from 1,560 cm^−1^ to 1,653 cm^−1^. The C-O stretching vibrations of carboxyl adS = O groups range from 1,051 cm^−1^ to 1,074 cm^−1^. The -SO_3_ groups range from 1,384 cm^−1^ to 1,401 cm^−1^. Lastly, the halogen or alkene groups range from 547 cm^−1^ to 560 cm^−1^. According to the analysis, we observed pronounced asymmetrical stretching bands and numerous shifts in the adsorption peaks among the wild-type, XT1-2-2-::APS, and XT1-2-2-::SiR strains. The overexpression of *cys*H and *cys*J significantly influenced the functionality of certain chemical bonds within these strains, as revealed by FT-IR analysis, resulting in alterations to their adsorption capacity for Cd^2+^.

### XPS difference analysis

**Fig 7:**
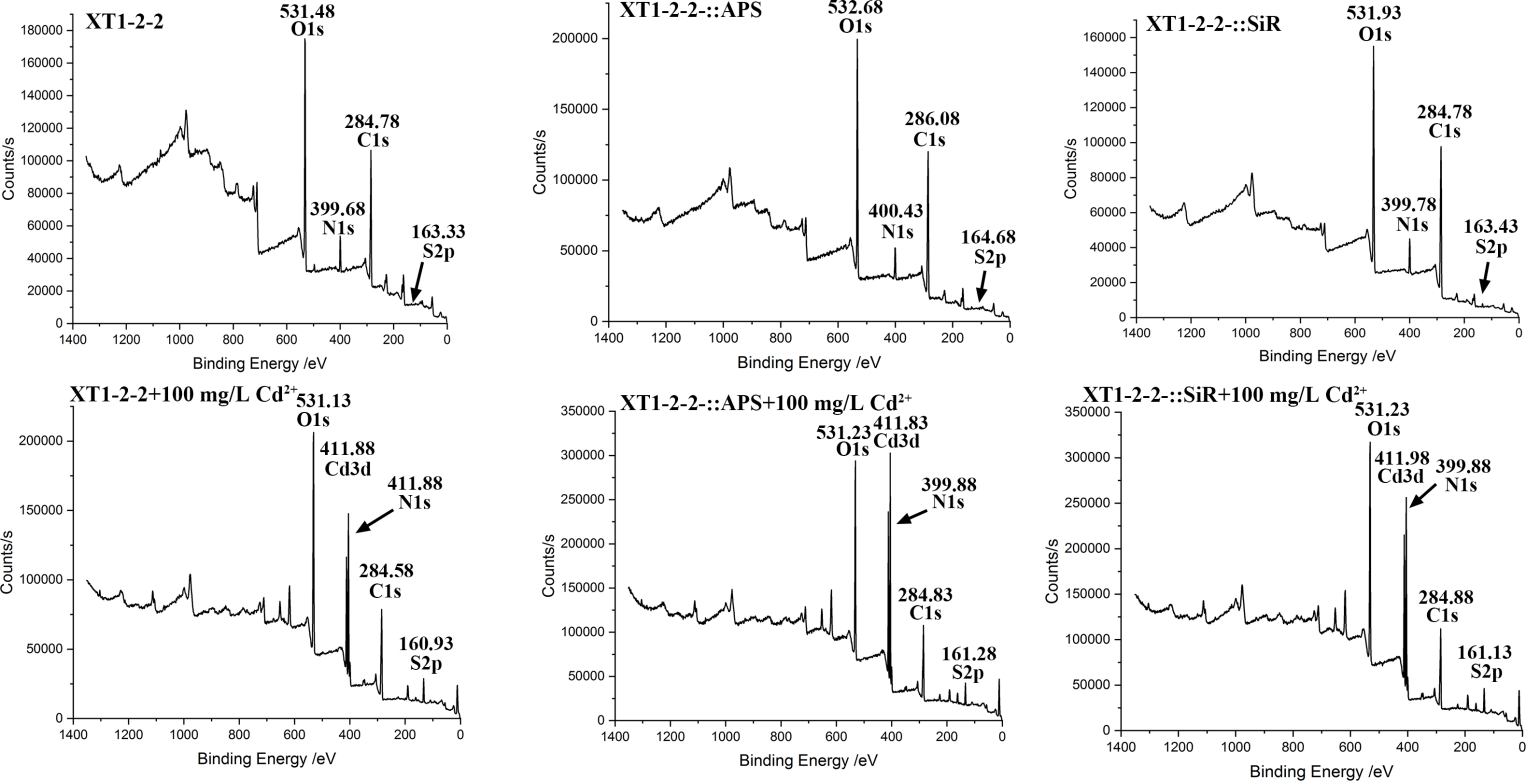
Analysis of X-ray photoelectron spectroscopy (XPS) spectra for the wild-type, XT1-2-2-::APS, and XT1-2-2-::SiR strains.

XPS analysis was performed to identify the flocculated sediments present either within or on the surface of the strains, with the results depicted in Fig. 7. The findings revealed two distinct peaks at 160.93 eV and 411.88 eV for the XT1-2-2 strain, at 161.28 eV and 411.83 eV for the XT1-2-2-::APS strain, and at 161.13 eV and 411.98 eV for the XT1-2-2-::SiR strain, which were attributed to S_2p_ and Cd_3d_ transitions, respectively. These specific peaks indicate the presence of CdS. Both XT1-2-2-::APS and XT1-2-2-::SiR exhibited a peak at 411.83 eV, with relative abundances exceeding that of the wild-type strain. These results suggest that overexpression of *cys*H and *cys*J may enhance CdS formation and influence cadmium reduction processes.

### p-XRD difference analysis

**Fig 8:**
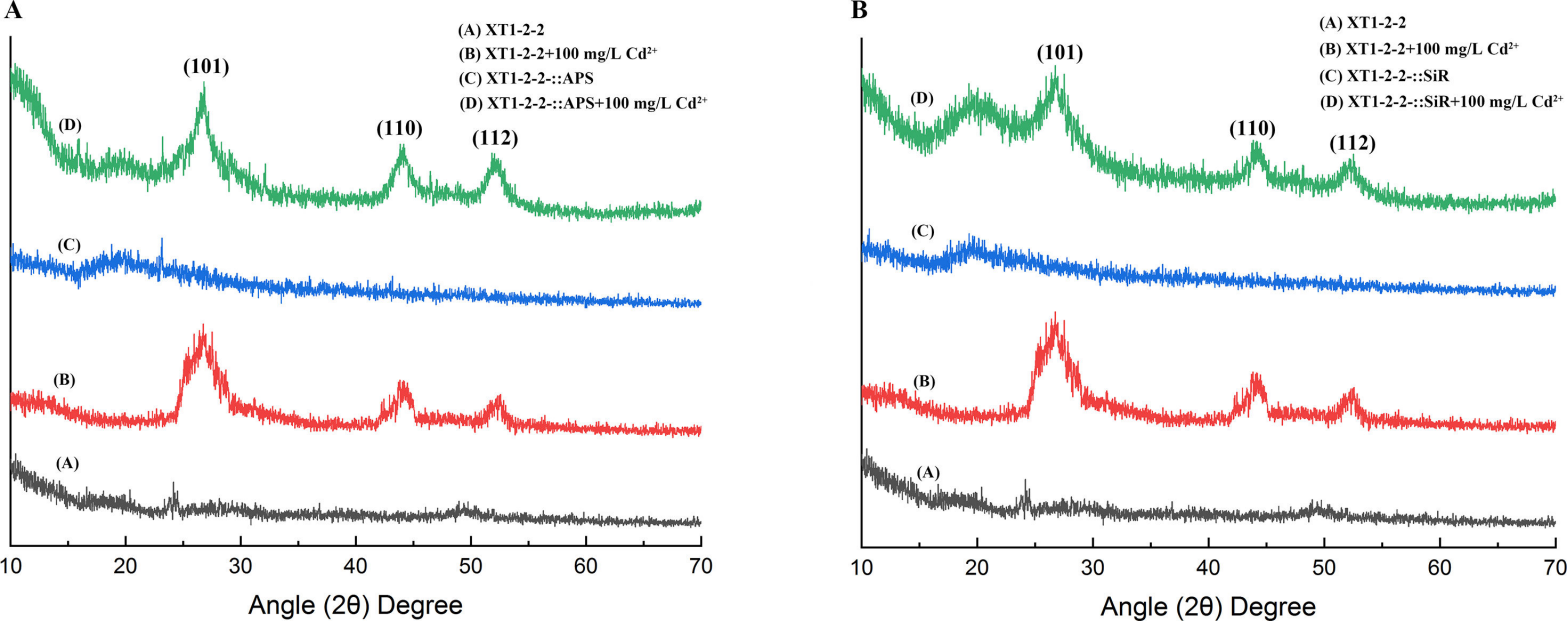
(**A**) Powder X-ray diffraction (p-XRD) analysis of the wild-type and XT1-2-2-::APS strains. (**B**) Powder X-ray diffraction (p-XRD) analysis of the wild-type and XT1-2-2-::SiR strains.

The crystal structures of flocculated sediments formed in the wild-type, XT1-2-2-::APS, and XT1-2-2-::SiR strains were examined through p-XRD. The crystalline peaks of flocculated sediment from the wild-type, XT1-2-2-::APS, and XT1-2-2-::SiR strains are illustrated in Fig. 8. The three prominent peaks observed at 26.55°, 44.06°, and 52.25° correspond to the Miller indices (101), (110), and (112), respectively, which are characteristic of CdS. The presence of CdS in the samples is evidenced by these three characteristic peaks. The results demonstrated that significant peaks of CdS were present in the wild-type, XT1-2-2-::APS, and XT1-2-2-::SiR strains cultured with 100 mg/L Cd^2+^. In contrast, no peaks were observed in the strains without the addition of Cd^2+^. These findings indicate that CdS formation occurred in the wild-type, XT1-2-2-::APS, and XT1-2-2-::SiR strains.

### Microcosm experiments

#### Bioavailability of cadmium in rice

**Fig 9:**
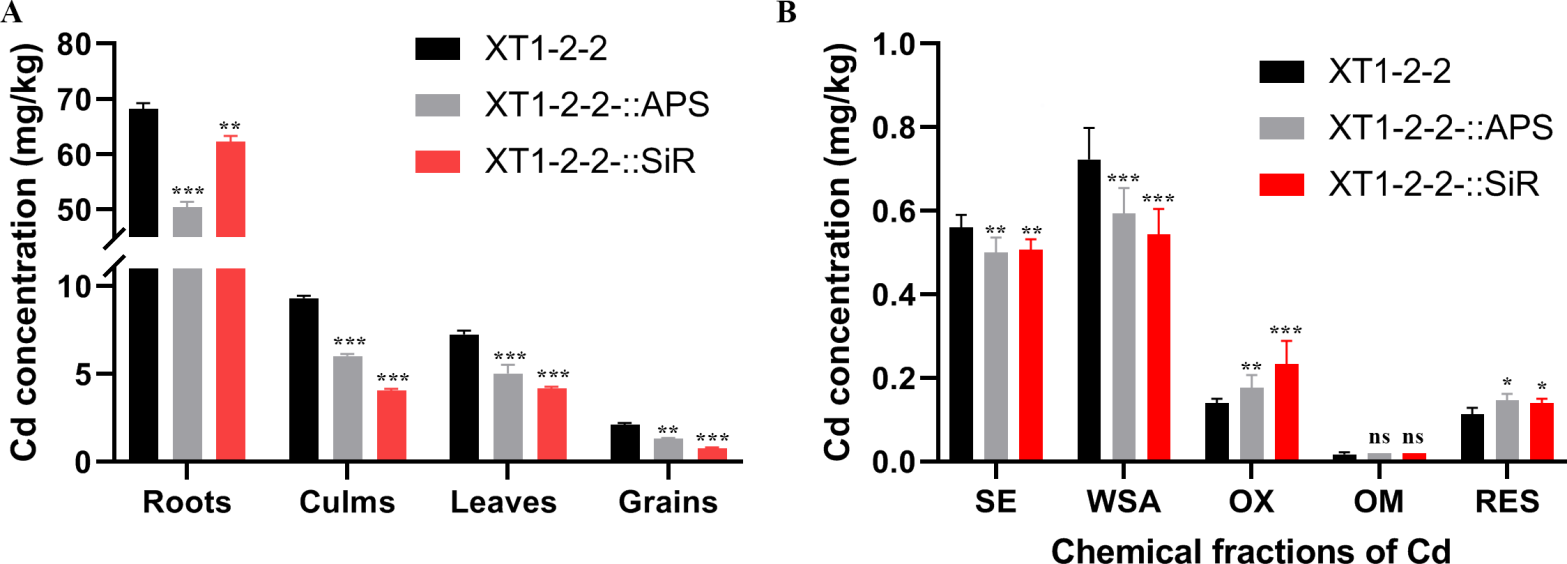
Microcosm experiments involving the wild-type, XT1-2-2-::APS, and XT1-2-2-::SiR strains were conducted. (**A**) The impact of these strains on cadmium concentrations in various tissues of rice plants is presented. Data are expressed as means ± standard deviations. (**B**) Cadmium speciation in soil inoculated with the respective strains is illustrated. Data are shown as means ± standard deviations for the following fractions: water-soluble plus exchangeable fractions (SE), carbonate-bound fractions (WSA), Fe/Mn oxide-bound fractions (OX), organic matter-bound fractions (OM), and residual fractions (RES). Standard errors from three replicates are indicated by error bars. Asterisks (*, **, ***) denote significance levels of *P* < 0.05, *P* < 0.01, and *P* < 0.001, respectively.

The cadmium concentration results in the roots, culms, leaves, and grains from microcosm experiments under various treatments are presented in Fig. 9A. The concentrations of cadmium in the roots, culms, leaves, and grains of the XT1-2-2-::APS strain exhibited reductions of 26.09%, 35.27%, 30.52%, and 36.49%, respectively. Similarly, the concentrations of cadmium in the roots, culms, leaves, and grains of the XT1-2-2-::SiR strain showed decreases of 8.63%, 56.45%, 42.54%, and 62.56%, respectively. The cadmium concentrations detected in the roots, culms, leaves, and grains inoculated with the XT1-2-2-::APS and XT1-2-2-::SiR strains were significantly diminished compared to those recorded in the wild-type strain. The overexpression of APS reductase and SiR reductase enhanced the efficiency of sulfur metabolism, thereby promoting the formation of H_2_S in the strains. Furthermore, utilizing these XT1-2-2-::APS and XT1-2-2-::SiR strains resulted in a reduction of cadmium concentration in rice grains. Consequently, this also improved the quality of the produced rice, bringing it closer to China’s cadmium limit standard for rice.

#### Antioxidant enzyme activities in leaves

The activities of antioxidant enzymes in leaves at the mature stage, inoculated with the XT1-2-2, XT1-2-2-::APS, and XT1-2-2-::SiR strains, were assessed (Fig. S7). The six enzymes utilized in this study comprise superoxide dismutase (SOD), glutathione peroxidase (GSH), L-ascorbic acid (Vc), catalase (CAT), malate dehydrogenase (MDA), and peroxidase (POD). The overexpression of *cys*H and *cys*J resulted in a decrease in the levels of six specific enzymes within the leaves. The reduction rates for SOD, GSH, Vc, CAT, MDA, and POD in the XT1-2-2-::APS strain compared to wild-type strain were 29.24%, 18.65%, 8.23%, 13.04%, 3.71%, and 12.67%, respectively. In contrast, the reduction rates for SOD, GSH, Vc, CAT, MDA, and POD in the XT1-2-2-::SiR strain compared to wild-type strain were recorded at 17.92%, 24.73%, 12.79%, 16.94%, 10.21%, and 20.88%, respectively.

#### Analysis of soil cadmium bioavailability differences

To assess the efficacy and potential risks associated with soil remediation using the wild-type, XT1-2-2-::APS, and XT1-2-2-::SiR strains, we analyzed the changes in the geochemical forms of soil cadmium. The results are presented in Fig. 9B. For paddy soils inoculated with the XT1-2-2 strain, the water-soluble plus exchangeable (SE) cadmium concentration was measured at 0.56 mg/kg, accounting for nearly 12% of the total cadmium content. This finding indicates a high bioavailability of cadmium in the soil. In contrast, for paddy soils treated with the XT1-2-2-::APS and XT1-2-2-::SiR strains, both soluble plus exchangeable (SE) and carbonate-bound (WSA) cadmium levels were significantly reduced. However, there was an increase in Fe/Mn-bound (OX), organic matter-bound (OM), and residual (RES) cadmium fractions. Compared to the wild-type strain, the soluble plus exchangeable (SE) cadmium levels in the XT1-2-2-::APS and XT1-2-2-::SiR strains were significantly reduced by 10.71% (*P* < 0.05) and 8.93% (*P* < 0.05), respectively. Additionally, carbonate-bound (WSA) cadmium decreased by 18.06% (*P* < 0.01) and 25% (*P* < 0.01). In contrast, Fe/Mn oxide-bound (OX) cadmium increased by 28.57% (*P* < 0.05) and 64.29% (*P* < 0.01), while residual (RES) cadmium increased by 36.36% (*P* < 0.01) and 27.27% (*P* < 0.01), respectively. Furthermore, there was no significant difference observed in organic matter-bound (OM) cadmium levels between the wild-type strain and the XT1-2-2-::APS or XT1-2-2-::SiR strains.

## DISCUSSION

Although heavy metals in soil can inhibit the growth of certain soil microorganisms, there exists a class of functional bacteria in nature capable of remediating cadmium-contaminated soil (41). In our previous research (42), we discovered that the *Citrobacter* sp. XT1-2-2 strain effectively promotes rice growth and plays a significant role in the remediation of cadmium-contaminated soil. The mechanism by which *Citrobacter* sp. XT1-2-2 removes cadmium primarily involves biological adsorption and biological precipitation. Biological precipitation occurs through the synergistic action of various metabolic pathways and enzymes within the bacteria, effectively enriching and precipitating heavy metals. The interaction between sulfur ions, generated from H_2_S in the sulfur metabolism pathway, and free cadmium ions present in soil leads to the formation of residual CdS. This process constitutes a critical mechanism underlying the cadmium tolerance and immobilization capability of the XT1-2-2 strain. Through genome sequencing of the XT1-2-2 strain, functional annotation of key regulatory genes, and KEGG metabolic pathway analysis, we identified that *cys* family genes play a significant role in regulating the sulfate metabolism pathway of *Citrobacter* sp. XT1-2-2. The APS reductase encoded by *cys*H is a key rate-limiting enzyme in the sulfate metabolism pathway, playing a crucial role in the conversion of SO_4_^2−^ to SO_3_^2−^. Additionally, *cys*J, which encodes SiR reductase, is essential for the transformation of SO_3_^2−^ to S^2−^. These enzymes can work synergistically with other bacterial enzymes to ultimately produce H_2_S. Previously, research validated the physical and chemical characteristics, as well as the fundamental functions, of APS reductase through *in vitro* induced expression and gene knockout experiments (24). In this study, we further investigated the working mechanisms of APS reductase and SiR reductase, along with their critical regulatory roles in cadmium reduction within bacterial strains, by overexpressing *cys*H and *cys*J (Fig. S8 and S9). The expression levels of the key genes *cys*H and *cys*J in the sulfate assimilation pathway were significantly enhanced through genetic engineering, leading to the successful construction of engineered strains with high sulfur metabolic activity.

**Fig 10:**
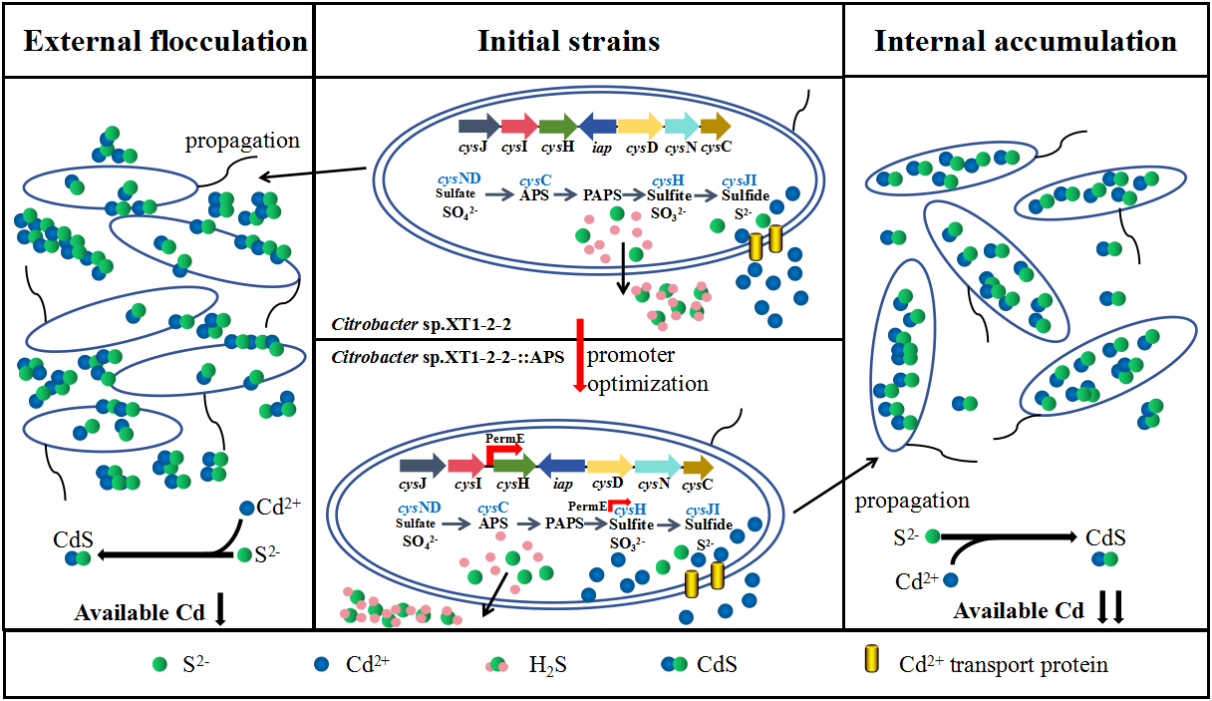
A conceptual model illustrating the sulfate metabolic pathways involved in the formation of CdS within *Citrobacter* sp. XT1-2-2-::APS.

The differences in cadmium biological adsorption and precipitation among the wild-type strain, XT1-2-2-::APS, and XT1-2-2-::SiR strains were examined using TEM. Observations revealed a significant accumulation of black particles in both the XT1-2-2-::APS and XT1-2-2-::SiR strains. In contrast, the wild-type strain exhibited simultaneous extracellular and intracellular absorption, with the majority of sediment accumulating outside the bacterial cells. The experimental results indicated that the interaction between bacterial surface functional groups and Cd^2+^ prompted the secretion of extracellular polysaccharides, iron carriers, organic acids, and S^2−^. This process was facilitated by the combined action of sulfate metabolism enzymes and other proteins involved in related metabolic pathways within the bacteria. In comparison to the wild-type strain, both the XT1-2-2-::APS and XT1-2-2-::SiR strains may produce more hydrogen sulfide and facilitate a greater extent of CdS biosynthesis within the bacteria (Fig. 10; Fig. S10). The findings also indicated that the overexpression of *cys*H or *cys*J influenced the localization of CdS formation within the bacterial cells. The APS reductase and SiR reductase in *Citrobacter* sp. XT1-2-2 plays a crucial role in the sulfate metabolism pathway, thereby providing a robust theoretical foundation for the development of novel bioremediation technologies based on synthetic biology.

The bioavailability of cadmium in soil directly influences its enrichment in rice. Soluble exchange (SE) and carbonate-bound (WSA), as highly active components, are readily absorbed by plants due to their high reactivity. In contrast, iron and manganese oxide-bound (OX), organic matter-bound (OM), and residue form (RES) exhibit low bioavailability owing to their high stability. The results demonstrated that the engineered strains XT1-2-2-::APS and XT1-2-2-::SiR promoted the formation of CdS nanoparticles through the enhanced hydrogen sulfide biosynthesis pathway, facilitating the combination of S^2−^ produced by microbial metabolism with Cd^2+^ in soil. This process significantly altered the morphological distribution characteristics of cadmium. Compared with the wild-type strain, the XT1-2-2-::APS and XT1-2-2-::SiR strains effectively reduced the concentrations of highly active SE and WSA forms of cadmium in soil while promoting the transformation of cadmium into more stable OX and RES forms. However, they had no significant impact on OM-bound cadmium. This mechanism of converting active cadmium into inert components substantially decreased the accumulation of cadmium in rice tissues, indicating that microorganisms can regulate the environmental migration and bioavailability of cadmium by altering its chemical speciation. This discovery elucidates the critical mechanism by which functional microorganisms immobilize cadmium via sulfur metabolic pathways and provides a novel theoretical basis and research direction for the *in situ* stabilization strategy of cadmium pollution based on microbial regulation. Furthermore, it lays a scientific foundation for the development of bioremediation technologies targeting specific cadmium binding forms.

Biofilm formation represents a protective mode of growth that enables bacteria to disperse and colonize new niches while also allowing them to survive harsh environments. Biofilm formation provides a certain degree of stability within the growth environment. Furthermore, it offers protection against a wide array of environmental challenges, including metal toxicity, acid exposure, ultraviolet radiation, dehydration, salinity, and antimicrobial agents (43). In this study, the overexpression of *cys*H and *cys*J led to varying degrees of enhanced bacterial biofilm formation, thereby improving the adaptability of *Citrobacter* sp. XT1-2-2 to cadmium-rich environments. The XT1-2-2-::APS strain demonstrated the highest rate of cell aggregation. The enhanced capacity for cell aggregation observed in the XT1-2-2-::APS strain may be attributed to its elevated expression level of *cys*H. The impact of the overexpression of *cys*H and *cys*J on biofilm production has been reported for the first time.

To investigate the impact of the wild-type and XT1-2-2-::APS and XT1-2-2-::SiR strains on cadmium uptake by grains, rice growth, and the bioavailability of cadmium in soil, we conducted microcosm experiments. The results indicated that rice plants subjected to the XT1-2-2-::APS and XT1-2-2-::SiR strains demonstrated significantly improved growth (data not shown) and exhibited lower cadmium content compared to other treatments. These results indicate that the elevated expression levels of APS reductase and SiR reductase enhance the efficiency of sulfur metabolism, facilitate related enzymatic reactions, and consequently influence the immobilization of cadmium by bacteria in soil as well as the growth of rice. These results also indicate that the potential of microbial environmental remediation can be substantially enhanced through precise metabolic pathway optimization.

The activities of antioxidant enzymes in leaves at the mature stage, inoculated with the wild-type, XT1-2-2::APS, and XT1-2-2::SiR strains, were found to be reduced to a certain extent compared to those in wild-type strain (Fig. S7). Microorganisms can improve the physiological condition of rice plants by reducing cadmium absorption and inhibiting the generation of reactive oxygen species (17). As a result, leaves may not necessitate high levels of antioxidant enzyme activity to mitigate cadmium stress, which leads to a decrease in the activity of these enzymes within the leaves.

Based on the experimental findings of this study, we developed a conceptual model that illustrated the relationship between the optimization of the APS reductase and CdS accumulation during the culture process, as depicted in Fig. 10. The majority of CdS produced by XT1-2-2-::APS and XT1-2-2-::SiR was found to accumulate within the bacterial cells, whereas in the wild-type strain, CdS primarily manifested as external flocculation. The results clearly demonstrated that the overexpression of *cys*H and *cys*J not only enhanced efficiency of sulfur metabolism but also significantly improved cadmium immobilization. The sulfate metabolism pathway encompasses numerous genes from the *cys* family, and further analysis is required to investigate their additional functions and effects.

The field application of engineered *Citrobacter* strains for cadmium remediation requires a comprehensive evaluation of ecological interactions with native soil microbiomes. While enhanced H_2_S production facilitates efficient CdS biomineralization, it also presents risks, such as the inhibition of anaerobic microbiota, plant phytotoxicity, and disruption of indigenous communities through competitive exclusion or horizontal gene transfer (*cys*H/*cys*J). To address these concerns, field deployment will incorporate: (1) optimized inoculation densities to limit H_2_S accumulation; (2) exclusion from waterlogged environments to prevent sulfide retention; and (3) phased application in contamination hotspots. Ecological impacts will be meticulously monitored through real-time tracking of H_2_S levels, high-throughput sequencing to assess shifts in microbial diversity, and metagenomic surveillance to evaluate community resilience and the dominance of engineered strains. These strategies collectively aim to balance remediation efficacy by reducing cadmium bioavailability with long-term microecological safety and ecosystem stability.

### Conclusions

This study highlights the critical role of the *cys*H and *cys*J genes in the sulfur-mediated cadmium detoxification pathway of *Citrobacter* sp. XT1-2-2 strain. The targeted overexpression of these genes significantly increased hydrogen sulfide production, thereby facilitating the intracellular biosynthesis of CdS nanoparticles and enhancing cadmium immobilization efficiency. Physicochemical characterizations, including TEM, FT-IR, XPS, and p-XRD, have confirmed the structural and metabolic modifications that underlie these phenotypic enhancements. Microcosm experiments further substantiated the practical efficacy of the engineered strains, demonstrating a significant reduction in cadmium accumulation across plant tissues when compared to the wild-type strain. These findings not only enhance our understanding of microbial sulfur metabolism in the context of heavy metal detoxification but also establish a genetic engineering framework for optimizing bacterial-assisted bioremediation strategies. Future research should prioritize the exploration of multigene co-expression systems to enhance sulfide production further. This should be accompanied by field trials aimed at evaluating ecological impacts and remediation efficacy under real-world conditions. Such advancements will facilitate the translation of genetic engineering innovations into sustainable environmental biotechnologies within contaminated agricultural ecosystems.

### Environmental implication

Cadmium pollution has emerged as a significant environmental concern, resulting in increased toxicity and posing threats to food safety and public health within the soil-crop system and the broader food chain. Microbial remediation shows considerable potential for addressing cadmium-contaminated soils; however, the genetic basis underlying its sulfur-mediated bioremediation mechanisms remains inadequately understood. This study highlights the critical roles of the *cys*H and *cys*J genes in the sulfur-mediated cadmium detoxification pathway of *Citrobacter* sp. XT1-2-2 strain. The targeted overexpression of these genes significantly enhanced both sulfur metabolism efficiency and cadmium immobilization capacity. The findings from this study will provide a theoretical foundation for exploring novel bacterial-assisted techniques aimed at cadmium remediation.

## Data Availability

The *Citrobacter* sp. XT1-2-2 genome sequence data were deposited in NCBI with the accession number PRJNA836337. Data will be made available on request.

## SUPPLEMENTAL MATERIAL

Supplemental material aem.00856-25-s0001.docx Tables S1 and S2; Fig. S1 to S11.

ASM does not own the copyrights to Supplemental Material that may be linked to, or accessed through, an article. The authors have granted ASM a non-exclusive, world-wide license to publish the Supplemental Material files. Please contact the corresponding author directly for reuse.
