# Supplementary material for: Genetic manipulation of cysH/cysJ in Citrobacter sp. XT1-2-2 enhanced cadmium immobilization by regulating metabolic pathways
Source: Appl Environ Microbiol. 2025 Aug 21;91(9):e00856-25. doi: 10.1128/aem.00856-25 (PMC12442384; doi:10.1128/aem.00856-25)
Supplement: Supplemental material — Tables S1 and S2; Fig. S1 to S11. [file aem.00856-25-s0001.docx]

*Title page-Supporting materials*

****Genetic manipulation of *cys*H/*cys*J in *Citrobacter* sp. XT1-2-2 enhanced cadmium immobilization by regulating metabolic pathways****

Zhudong Liu^1,2,†^, Wei Cheng^1,2,†^, Yilu Li^1,2,†^, Shiping Shan^1,2,3,*^, Shandong Wu^1,2^, Xiaowu Wei^1,2^, Hua Yang^1,2^, Min Zhang^1,2^, Dongxia Du^1,2^

1 *Hunan Institute of Microbiology, Hunan Academy of Agricultural Sciences, Changsha, Hunan, 410009, China*

*2 Yuelushan Laboratory, Changsha, Hunan, 410128, China*

3 *Hunan Engineering and Technology Research Center of Agricultural Microbiology Application*, *Changsha, Hunan, 410009, China*

*Author for correspondence:

Shiping Shan ([ssp312@hotmail.com](mailto:ssp312@hotmail.com))

Current Address: Hunan Institute of Microbiology, Hunan Academy of Agricultural Sciences, Changsha, Hunan, 410009, China.

Phone: (+86) 731 88858945.

Fax: (+86) 731 85261072.

† These authors have contributed equally to this work.

The supporting information has 8 pages including 2 tables and 11 figures.

**Table S1. Primers, plasmids , nucleotide sequences of primers and strains used in this study**

|  | **Relative description** | **Sources** |
| --- | --- | --- |
| **Strains** |  |  |
| *E.coli* Top10 | Host for general cloning | Lab store |
| *E.coli* S17-1 | Donor strains for conjugation | Lab store |
| *Citrobacter* sp. XT1-2-2 | Functional bacteria with cadmium reducing activity;Wild type strain | Lab store |
| ::APS | Over-expression strain of APS | This work |
| ::SiR | Over-expression strain of SiR | This work |
| *E.coli* BL21 | Heterologous host of protein expression | Lab store |
| **Plasmids** |  |  |
| pBBR1MCS-2 | Wide host expression plasmid | Lab store |
| pBBR1MCS-PermE-*cys*H | Plasmid used for the construction of the APS over-expression strain | This work |
| pBBR1MCS-PermE-*cys*J | Plasmid used for the construction of the SiR over-expression strain | Lab store |
| **PCR Primers** |  |  |
| PermE-F | 5’-CCG***GAATTC***CTGGACTTCTAGAGCTAGCC-3’（*Eco*RI） |  |
| PermE-R | 5’-GCATGCCGGTCGACTCTA-3’ |  |
| PermE-APS-F | 5’-GATCCTCTAGAGTCGACCGGCATGCATGTCCGTACTTGATCTACA-3’ |  |
| PermE-APS-R | 5’-TGC***TCTAGA***TTACCCTTCGTGCAGCCCGC-3’（*Xba*I） |  |
| PermE-SiR-F | 5’-GATCCTCTAGAGTCGACCGGCATGCATGACGACACAGGCCCCACC-3’ |  |
| PermE-SiR-R | 5’-TGC***TCTAGA***TTAGTAGACATCTCGCTGATAA-3’（*Xba*I） |  |
| verifyAPS-F | 5’-CTGGACTTCTAGAGCTAGCC-3’ |  |
| verifyAPS-R | 5’-TTACCCTTCGTGCAGCCCGC-3’ |  |
| verifySiR-F | 5’-CTGGACTTCTAGAGCTAGCC-3’ |  |
| verifySiR-R | 5’-TTAGTAGACATCTCGCTGATAA-3’ |  |

Note: Restriction enzyme sites were italic and bold, overlaping sequences were underlined.

**Table S2. Main physical and chemical properties of the paddy soil**

**used in the microcosm experiments**

| **pH** | **CEC (cmol/kg)** | 1. **M (g/kg)** | **P Available (mg/kg)** | **K Available (mg/kg)** | **Ammonium Nitrogen (mg/kg)** | **Total(mg/kg)** | | | | |
| --- | --- | --- | --- | --- | --- | --- | --- | --- | --- | --- |
|  |  |  |  |  |  | **Cd** | **As** | **Pb** | **Hg** | **Cr** |
| **5.3** | **11.3** | **38.6** | **3.9** | **176** | **25.2** | **0.39** | **15.6** | **35.4** | **0.15** | **77.6** |

**Note: CEC and OM refers the abbreviation of cation exchange capacity and organic matter.**


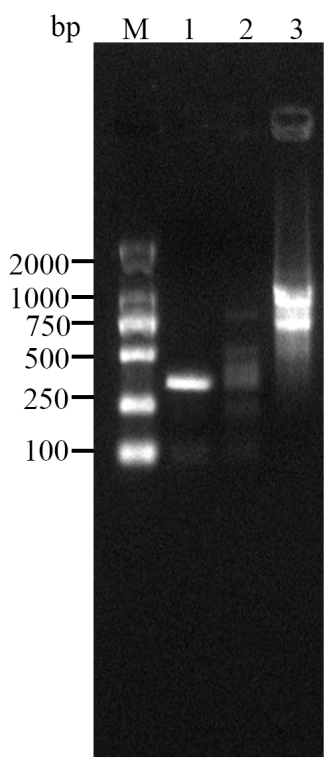


Fig.S1. 1. PCR analysis of the PermE promoter fragment. 2. PCR analysis of the *cys*H fragment. 3. PCR analysis of the combined PermE and *cys*H fragments.


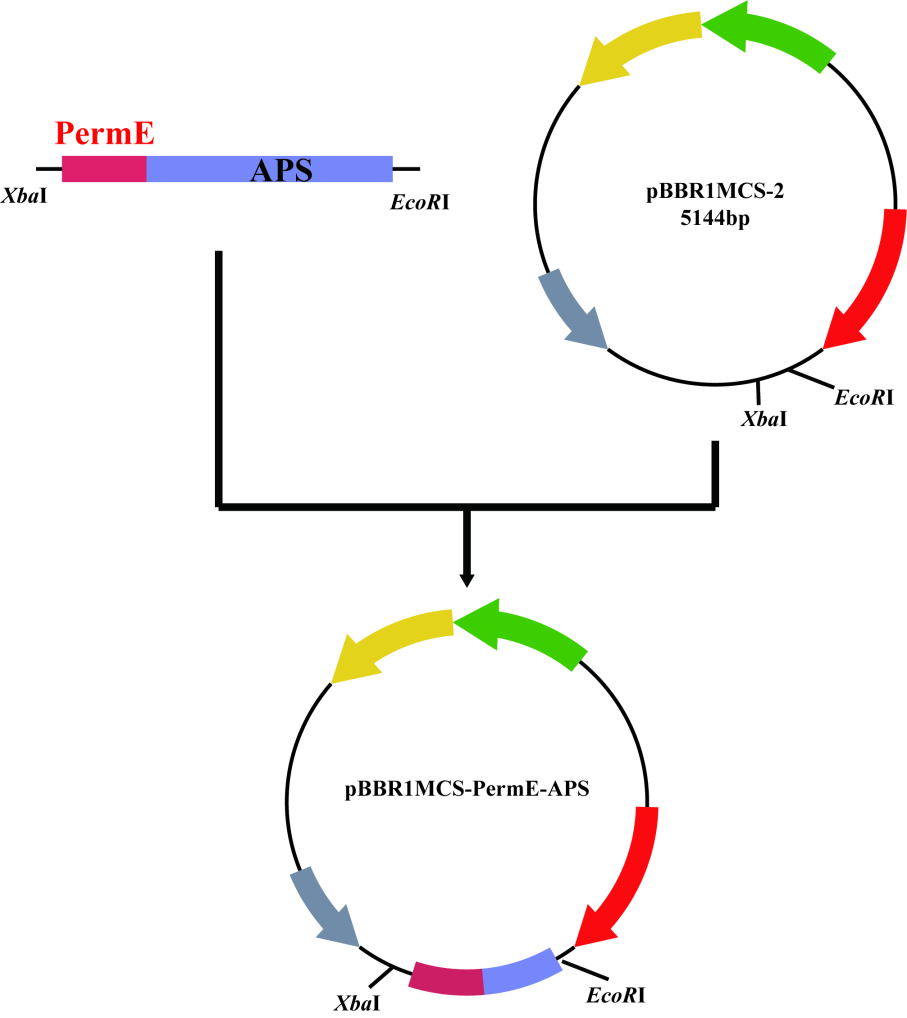


Fig.S2. The construction of pBBR1MCS-PermE-APS.


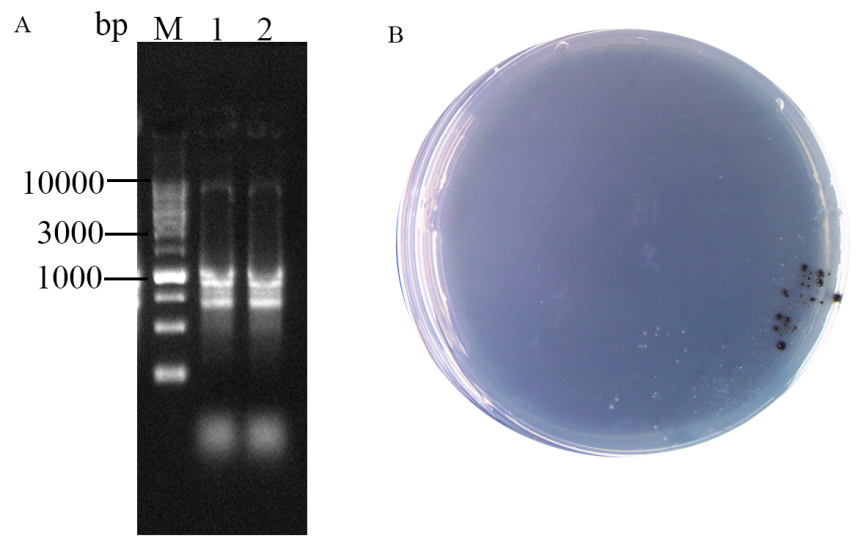


Fig.S3. A. Validation of pBBR1MCS-PermE-*cys*H through agarose gel electrophoresis following digestion with *Xba*I/*EcoR*I. B. Screening of positive transformants for the XT1-2-2-::APS.


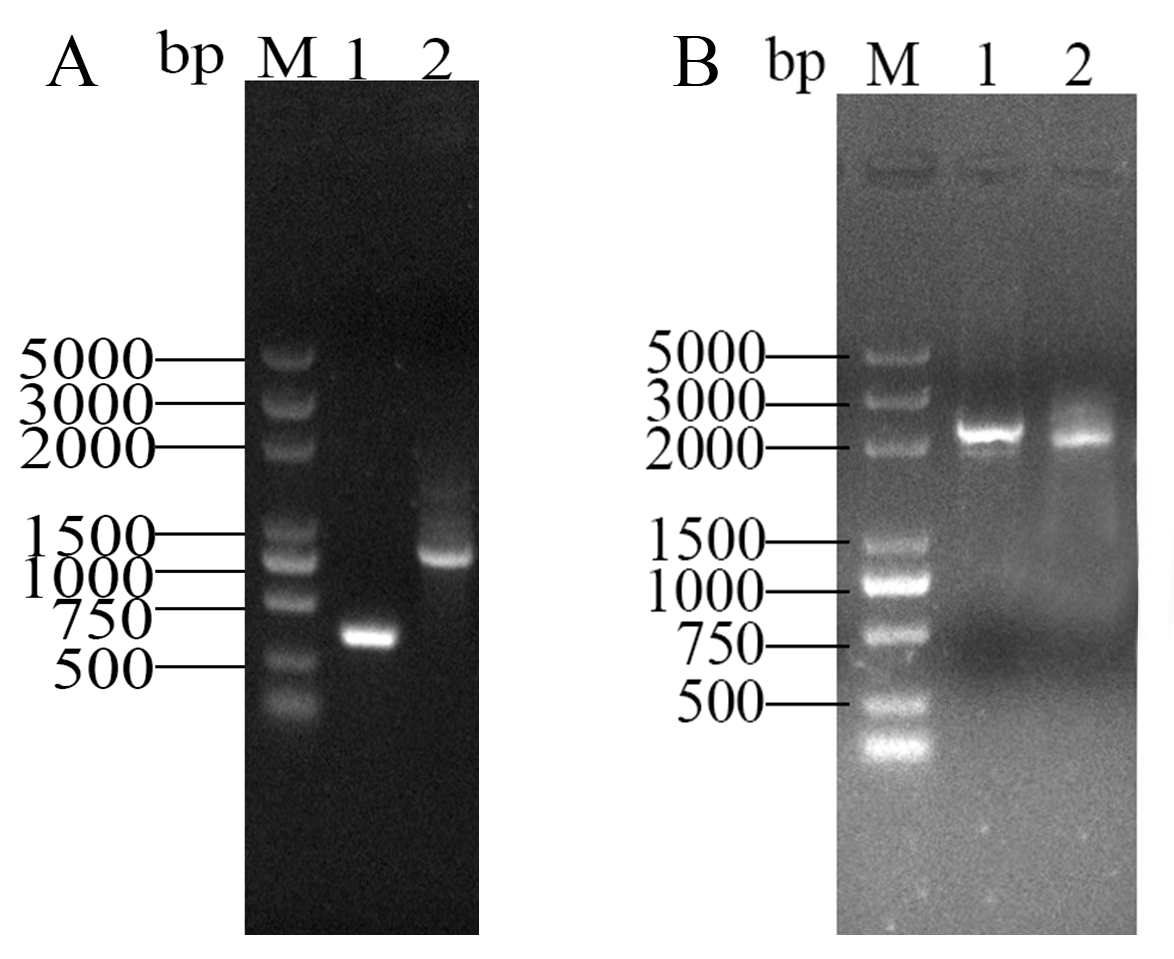


Fig.S4. A: 1. PCR analysis of the PermE promoter fragment; 2. PCR analysis of the *cys*J fragment. B: 1-2. Combined PCR analysis of PermE and *cys*J fragments.


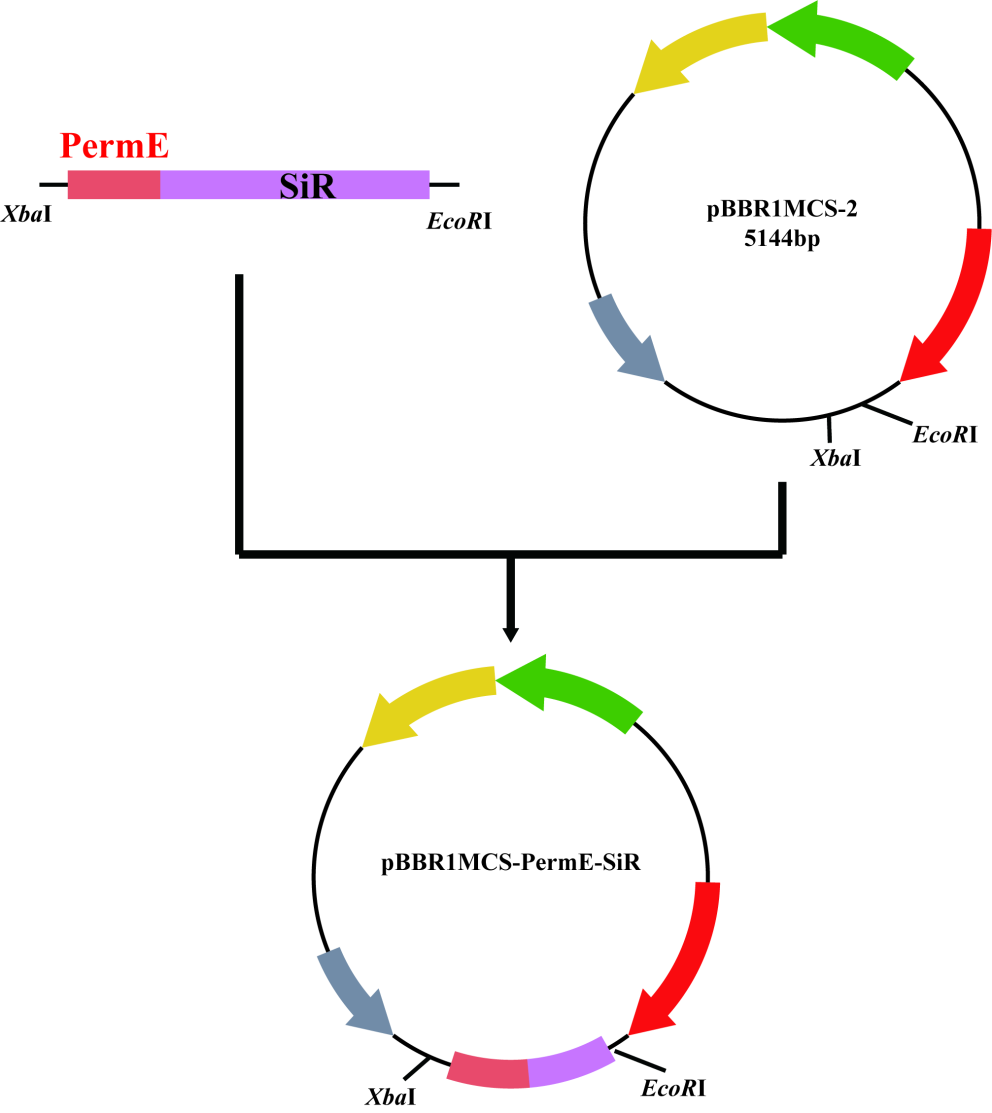


Fig.S5. The construction of pBBR1MCS-PermE-SiR.


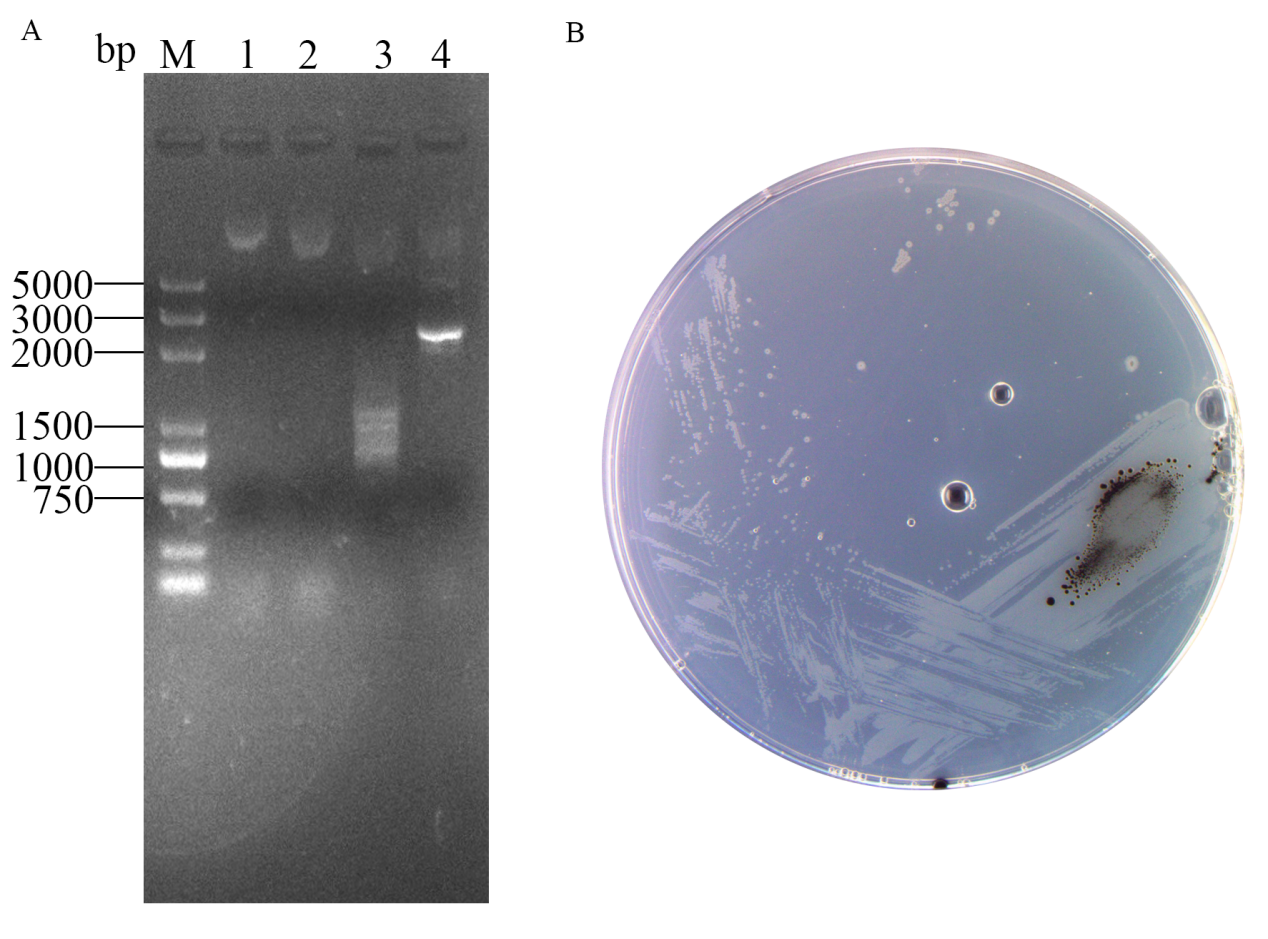


Fig.S6. A: 1-2. Extraction and detection of recombinant expression vector plasmids; 3. Double enzyme digestion of pBBR1MCS-2; 4. Validation of pBBR1MCS-PermE-*cys*J digested by *Xba*I/*EcoR*I through agarose gel electrophoresis; B: Screening for positive transformants of the XT1-2-2-::SiR.


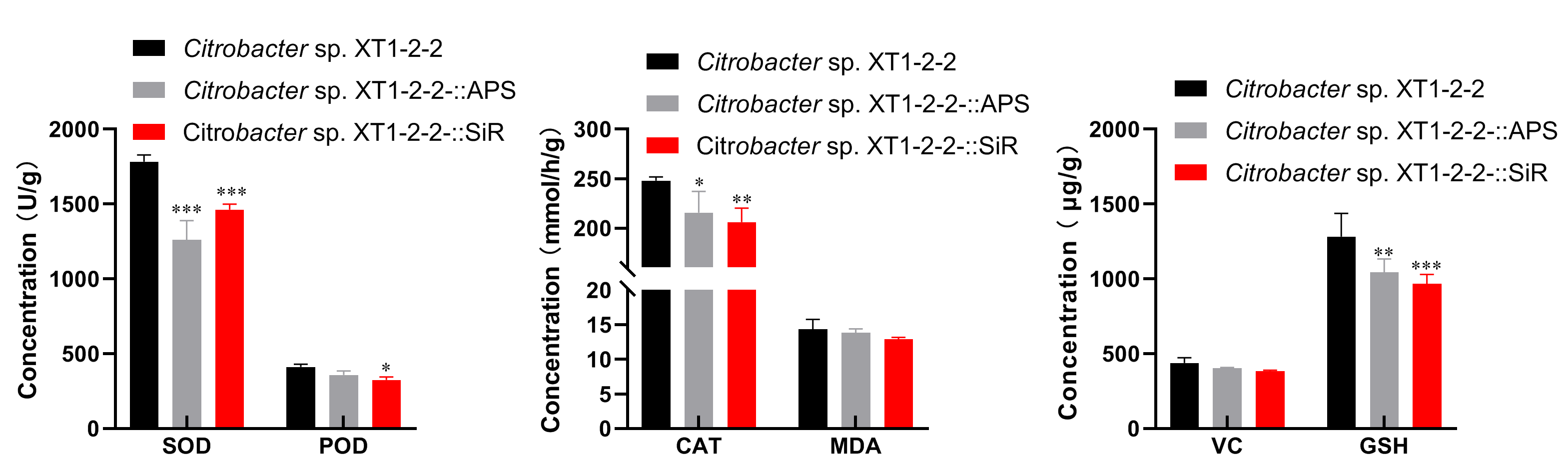


Fig.S7. The enzymatic activities of six enzymes in leaves at the mature stage, which were inoculated with the wild-type, XT1-2-2::APS, and XT1-2-2::SiR strains.


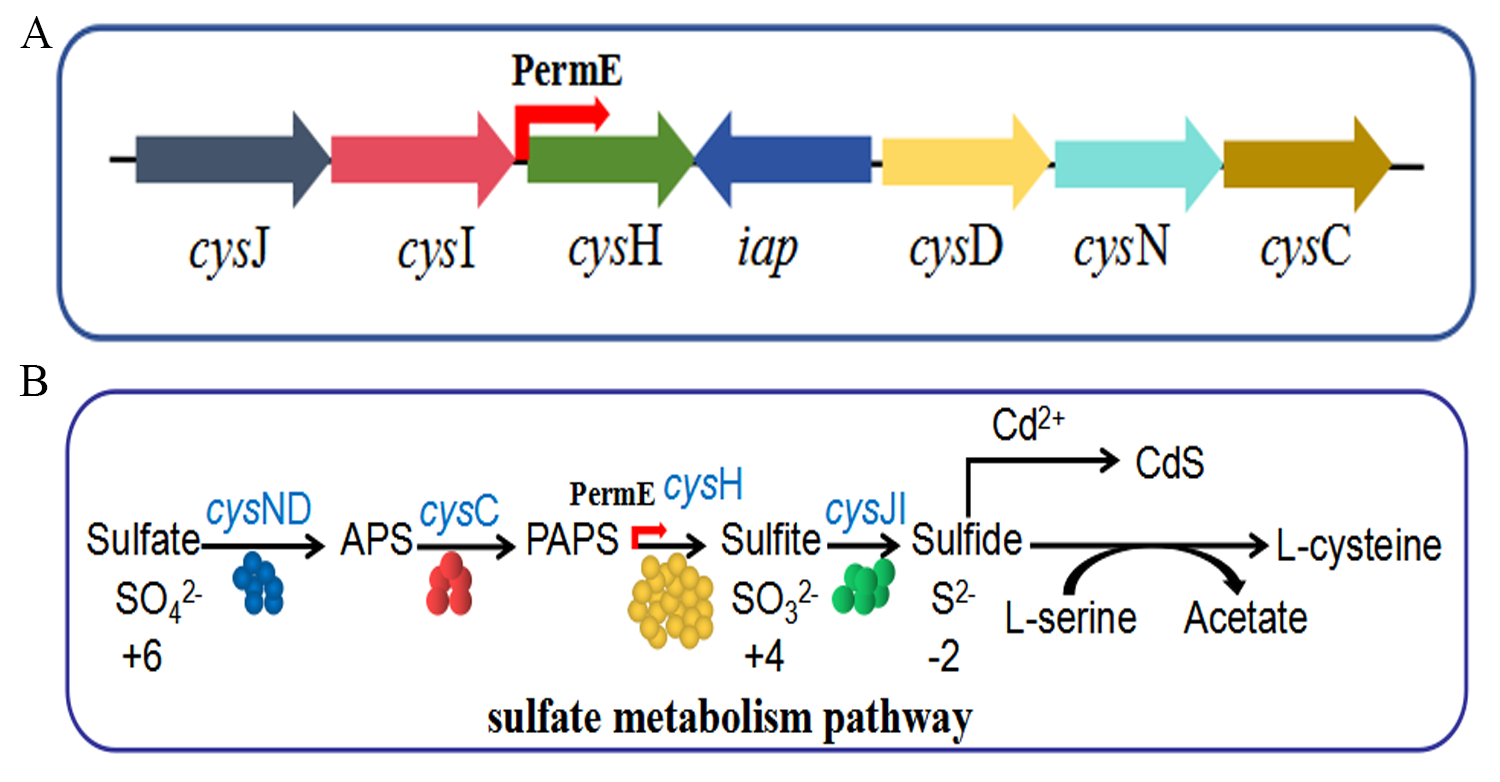


Fig.S8. A. The construction of a strong Erythromycin promoter (PermE) for *cys*H;

B. The sulfate metabolism pathway enhanced by the over-expression of *cys*H.


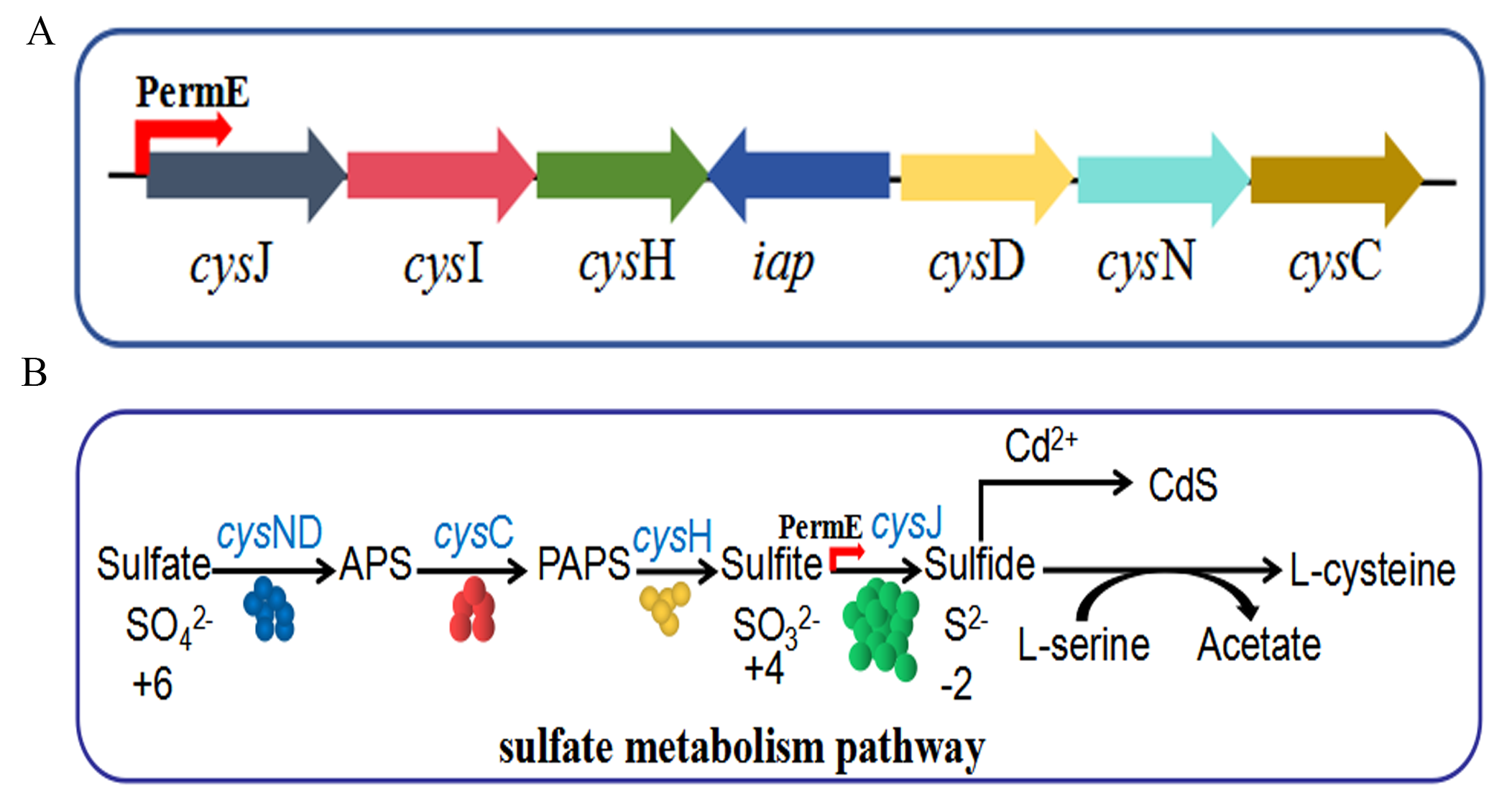


Fig.S9. A. The development of a robust Erythromycin promoter (PermE) for *cys*J;

1. The sulfate metabolism pathway enhanced by the over-expression of *cys*J.


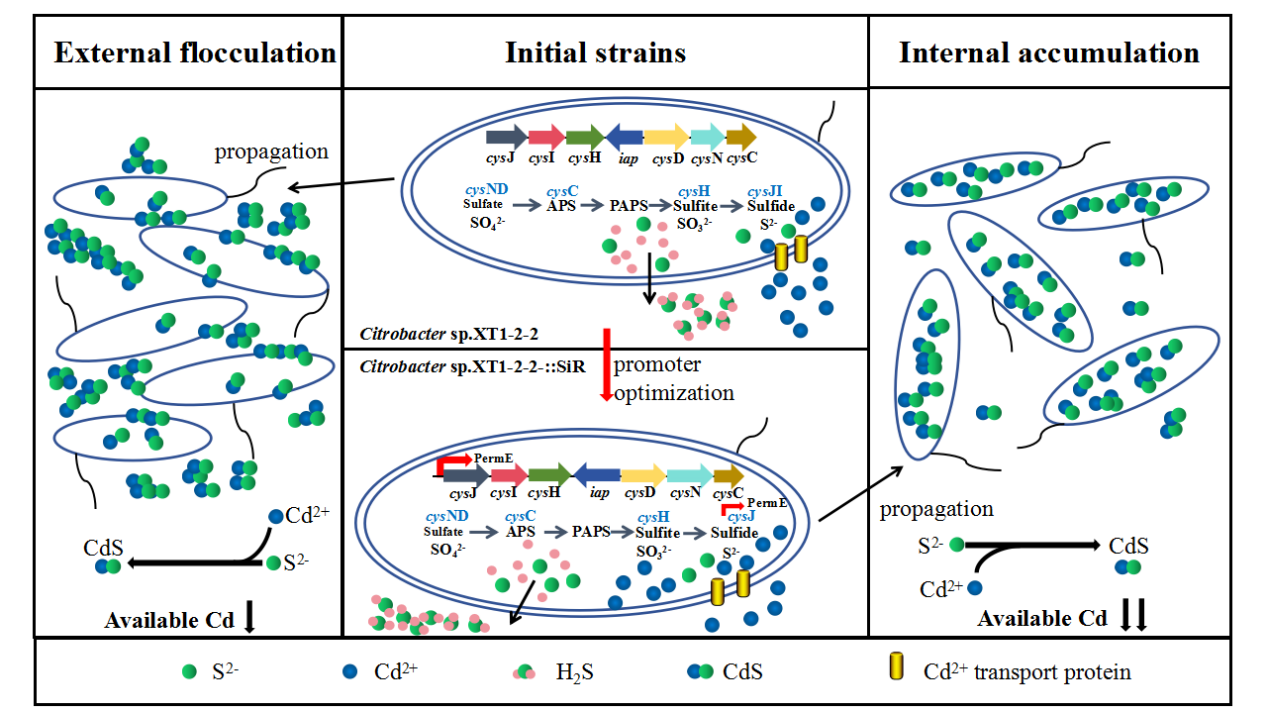


Fig.S10. A conceptual model illustrating the sulfate metabolic pathways involved in the formation of CdS in *Citrobacter* sp. XT1-2-2-::SiR.


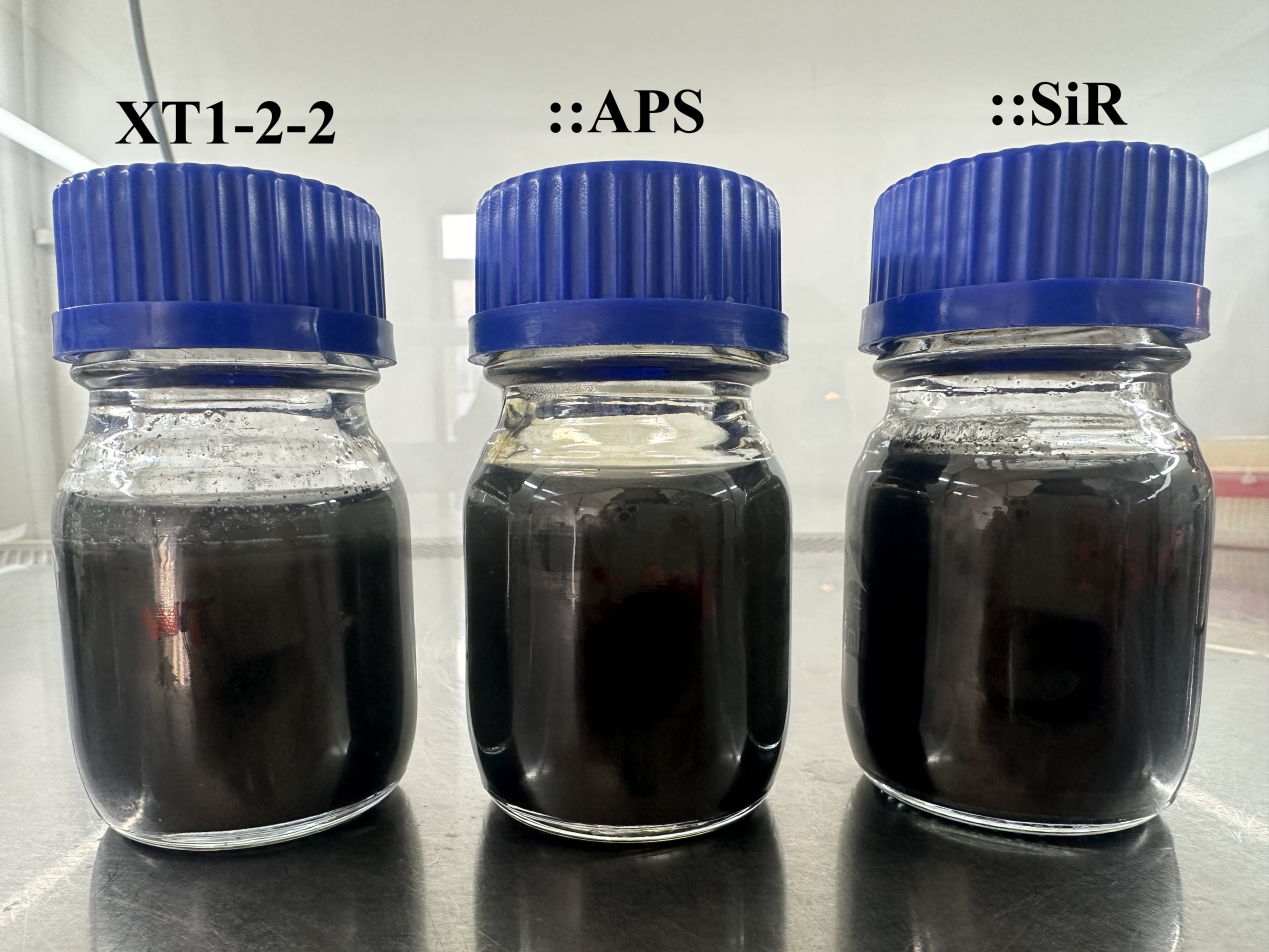


Fig.S11. The color differences among the wild-type, XT1-2-2::APS, and XT1-2-2::SiR strains in SRB medium containing Fe^2+^ are examined.
